# IRS assisted hybrid HAP UAV uplink NOMA networks: an interference aware optimization framework

**DOI:** 10.1038/s41598-026-46228-z

**Published:** 2026-04-18

**Authors:** Asma A. Alhashmi, Ahmed Badi Alshammari, Monir Abdullah, Abed Saif Ahmed Alghawli, Tareq M. Alkhaldi, Laith A. Darem, Imen B. Mohamed, Abdulbasit A. Darem

**Affiliations:** 1 Department of Computer Science, College of Science, Northern Border University, Arar, 73213 Saudi Arabia; 2 Department of Computer Science, College of Computing and Information Technology, Northern Border University, Rafha, 91911 Saudi Arabia; 3 Department of Computer Science and Artificial Intelligence, College of Computing and Information Technology, University of Bisha, Bisha, 61922 Saudi Arabia; 4 Department of Computer Science, College of Sciences and Humanities, Prince Sattam Bin Abdulaziz University, Al-Aflaj, Saudi Arabia; 5 Department of Educational Technologies, Imam Abdulrahman Bin Faisal University, Dammam, 4221 Saudi Arabia; 6 Department of Electrical Engineering, College of Engineering, Northern Border University, Arar, 73213 Saudi Arabia; 7 Center for Scientific Research and Entrepreneurship, Northern Border University, Arar, 73213 Saudi Arabia

**Keywords:** Engineering, Mathematics and computing

## Abstract

This paper investigates an intelligent reflecting surface (IRS)-assisted hybrid high-altitude platform (HAP) and unmanned aerial vehicle (UAV) uplink communication network employing non-orthogonal multiple access (NOMA). A unified uplink system model is developed that jointly captures UAV three-dimensional deployment, IRS-assisted composite channels, SIC-based uplink NOMA reception, and shared-spectrum cross-tier interference between HAP and UAV layers. To efficiently support dense uplink connectivity, a joint uplink sum-rate maximization problem is formulated under practical mobility, power, quality-of-service, and interference constraints, resulting in a highly non-convex mixed-integer optimization problem. A low-complexity block coordinate descent (BCD)-based framework is proposed to iteratively optimize UAV deployment, user association, uplink power allocation, and IRS phase shifts. The proposed framework is particularly well-suited for high-capacity data offloading and IoT-based safety monitoring in remote mining environments, supporting the digital transformation of the mining sector in alignment with Saudi Vision 2030. Extensive simulations demonstrate that the proposed framework significantly outperforms conventional aerial and terrestrial benchmarks. Specifically, compared to hybrid HAP–UAV uplink NOMA without IRS, the proposed design improves the uplink sum rate by up to 23.03% and by an average of 15.39% over K=10–100 simultaneously scheduled users. Relative to UAV-only and HAP-only IRS-assisted uplink schemes, gains of up to 43.80% and 59.01% are achieved, respectively, while effective improvement is observed over terrestrial uplink baselines under dense user loading. Moreover, IRS-assisted user association yields additional gains of up to 21.45% in sum rate, while outage probability is substantially reduced across the entire SINR range. These results confirm that IRS-assisted hybrid HAP–UAV uplink NOMA provides a scalable and reliable solution for future 6G-oriented dense uplink communication networks.

## Introduction and recent advancements

With the rapid evolution of next-generation wireless systems toward Sixth-Generation (6G) networks, there is a growing demand for ultra-high data rates, ubiquitous coverage, enhanced reliability, and low-latency communications^1^. These requirements become particularly critical in emergency and disaster scenarios, where terrestrial infrastructure may be partially or completely unavailable, as well as during large-scale temporary events that generate sudden traffic surges. In this context, aerial communication platforms have emerged as a promising solution to provide fast, flexible, and resilient connectivity. High-Altitude Platforms (HAPs) offer wide-area coverage and favorable Line-of-Sight (LoS) propagation conditions, making them well suited for supporting remote regions and post-disaster recovery operations^2,3^. Nevertheless, HAPs suffer from limited onboard resources and restricted processing capability, which may lead to congestion under dense user deployments or high traffic demand^2,4^. To alleviate these limitations, Unmanned Aerial Vehicles (UAVs) can be deployed as complementary low-altitude aerial nodes^5^. Owing to their mobility and flexible deployment, UAVs can provide localized, on-demand coverage and traffic offloading for HAP-assisted networks^6,7^. However, the coexistence of HAPs and UAVs over shared spectrum introduces new challenges related to cross-tier interference, user association, and resource allocation^8^. In parallel, Non-Orthogonal Multiple Access (NOMA) has been widely investigated as an effective uplink access technique to enhance spectral efficiency by allowing multiple users to transmit simultaneously over the same time–frequency resources and employing Successive Interference Cancellation (SIC) at multi-antenna receivers^9^. Moreover, the wireless propagation environment in urban and disaster-stricken areas is often severely affected by blockages and shadowing from buildings and other obstacles. Intelligent Reflecting Surfaces (IRSs) provides a promising means to reconfigure the radio environment by adaptively adjusting passive phase shifts to enhance desired signal paths and suppress interference^10–13^. Motivated by these advances, the integration of HAPs, UAVs, uplink NOMA, and IRS technology forms a powerful aerial networking paradigm. Such a hybrid architecture can simultaneously improve coverage, spectral efficiency, and robustness while enabling flexible interference management and resource optimization. Furthermore, this integrated framework is highly relevant for enhancing digital infrastructure in the mining sector, providing robust connectivity for geological exploration and real-time safety monitoring in line with the strategic objectives of Saudi Vision 2030. This makes it a strong candidate for future 6G-oriented emergency and high-density communication scenarios^14^.

### Recent advancements

Recent research has extensively explored aerial communication architectures based on HAPs and UAVs, and their hybrid integration with emerging technologies such as IRS^15^. In the following, we review representative works in HAP-based systems, UAV–HAP integration, and hybrid aerial networks, highlighting their achievements and remaining challenges.

#### Advancements in HAPs

HAPs have been recognized as a key enabler for large-scale aerial communication due to their wide coverage, stable LOS links, and suitability for long-duration missions^2,3,16,17^. Their role in disaster recovery and connectivity provisioning for underserved regions has been widely studied^8,18^. For instance^1^, proposed a gigabit-class HAP-based mobile communication system to ensure resilient wide-area coverage. Hybrid free-space optical and radio-frequency (Free-Space Optical (FSO)/Radio-Frequency (RF)) relaying architectures for HAPs were investigated in^2^, demonstrating improved reliability under varying channel conditions. In^19^, joint optimization of communication performance and flight operations was considered to enhance the energy efficiency of HAP deployments. Beyond communication, HAPs have also been incorporated into aerial edge computing frameworks. For example^3^, proposed a hierarchical aerial computing architecture combining HAPs and UAVs to support multi-access edge computing services for Internet of Things (IoT) applications. Similarly^20^, examined HAP-based sub-terahertz backhauling for massive Multiple-Input Multiple-Output (MIMO) systems. Despite these advances, HAP deployments face challenges such as high operational costs, regulatory constraints, weather sensitivity, and limited onboard processing capability, which motivate the integration of complementary aerial platforms such as UAVs^21,22^.

#### UAV integration with HAPs

UAVs have attracted significant attention due to their agility, rapid deployment, and dynamic adaptation of their positions to traffic demands. When integrated with HAPs, UAVs can serve as mobile access points or relays, enhancing spatial reuse and reducing service latency. For example^23^, studied full-duplex UAV-enabled IOT networks, while^6^ investigated secure access schemes for UAVs managed by HAPs using learning-based approaches. The integration of intelligent reflecting surfaces into UAV–HAP networks has further expanded their performance potential. Works such as^11^ and^12^ explored the use of IRS-assisted aerial platforms to improve spectral and energy efficiency. However, UAV- HAP coexistence over shared spectrum introduces severe interference coupling and increased resource allocation complexity. Uplink NOMA with SIC has been proposed in several studies to enhance spectrum utilization in aerial networks^9,24^. Nevertheless, most existing works either focus on simplified architectures or consider only partial optimization aspects, such as power control or deployment, without addressing a unified interference-aware design.

#### Hybrid integration of UAVs, HAPs, IRS, and uplink NOMA

The joint integration of UAVs, HAPs, IRS, and uplink NOMA has the potential to significantly enhance network performance by combining wide-area coverage, flexible deployment, environmental reconfiguration, and spectrum-efficient multiple access. Recent work has investigated partial combinations of these technologies across different contexts. For example^7^, studied learning-based frameworks in vertical heterogeneous aerial networks involving IRS-assisted UAVs and HAPs, while^13^ explored Hybrid Automatic Repeat Request (HARQ)-aided IRS-supported aerial backhaul systems. IRS-assisted hybrid FSO/RF aerial links were examined in^10^, demonstrating improved robustness under adverse channel conditions. In addition to maximizing spectral efficiency, ensuring high energy efficiency and low outage probability is a critical design objective for massive NOMA networks, particularly for ground devices with limited power budgets. Recent studies have demonstrated that Coordinated Direct and Relay Transmission (CDRT) frameworks using amplify-and-forward or decode-and-forward protocols can substantially enhance energy efficiency and minimize outage in NOMA systems, even under imperfect SIC conditions^25,26^. Building upon these insights, our proposed hybrid HAP–UAV framework explicitly incorporates network-wide transmit power budgets alongside minimum Signal-to-Interference-plus-Noise Ratio (SINR) thresholds to guarantee energy-efficient uplink transmission and strict Quality of Service (QoS) satisfaction. An emerging paradigm in IRS-assisted multiple access is IRS partitioning. Rather than optimizing a single, unified reflection matrix for all users simultaneously, partitioning strategies dynamically allocate discrete subsets of IRS elements to specific NOMA users, clusters, or signaling phases. As demonstrated in recent studies such as^27–29^, partitioning-enabled cooperative NOMA can significantly reduce optimization complexity, transforming difficult multidimensional searches into simpler integer-parameter allocations while simultaneously enhancing user fairness and energy efficiency. While our current framework employs a unified IRS reflection approach to maximize the aggregate uplink sum rate, integrating dynamic IRS element partitioning into the hybrid HAP–UAV architecture presents a highly promising avenue for reducing the computational overhead of the phase-shift optimization subproblem in future extensions. Despite these advances, existing studies typically optimize only subsets of the key design variables and often overlook the coupled uplink interactions induced by (i) shared-spectrum cross-tier interference, (ii) SIC-based intra-tier interference under uplink NOMA, and (iii) the joint dependence of composite IRS-assisted channels on UAV Three-Dimensional (3D) deployment and IRS phase shifts. Hence, based on the above discussion, a comprehensive interference-aware optimization framework for IRS-assisted HAP–UAV uplink networks with NOMA remains largely unexplored.

### Motivation and contributions

Motivated by the limitations of existing aerial communication architectures and the stringent requirements of emergency and high-density uplink scenarios, this work develops a rigorous and interference-aware uplink framework for IRS-assisted HAP–UAV integrated networks. In contrast to the predominantly downlink-oriented literature, reliable uplink transmission in multi-layer aerial systems is fundamentally constrained by three tightly coupled factors: heterogeneous aerial layers with distinct coverage characteristics, shared-spectrum cross-tier interference, and the strong dependence of uplink channel quality on UAV 3D deployment and IRS-controlled propagation paths. While HAPs provides stable wide-area connectivity, their long propagation distances and limited adaptability make them inefficient for serving dense or highly localized uplink traffic. The complementary deployment of UAVs as low-altitude access points improves spatial reuse and link quality, but introduces non-negligible cross-tier interference between HAP- and UAV-associated uplink users operating over the same spectrum, necessitating an explicit dual-layer uplink system formulation. In urban and disaster environments, uplink air-to-ground links are highly susceptible to blockage and shadowing. The incorporation of an IRS enables controllable signal reflection to enhance composite uplink channels and mitigate unfavorable propagation conditions. However, the achievable IRS gain is strongly coupled with user association, uplink power allocation, and UAV positioning, transforming the design problem into a geometry–propagation co-design challenge. To support massive connectivity and improve spectral efficiency, uplink NOMA with SIC is adopted at each aerial platform. Unlike downlink NOMA, uplink NOMA performance critically depends on SIC decoding order, residual intra-tier interference, and feasibility under shared-spectrum cross-tier interference and QOS constraints, which must be explicitly modeled to ensure practically achievable uplink rates. Despite these considerations, most existing studies address only partial combinations of HAPs, UAVs, IRSs, or uplink NOMA, often relying on fixed deployments, simplified interference assumptions, or downlink-centric designs. A unified uplink model that jointly captures UAV 3D deployment, IRS-assisted composite channels, SIC-based uplink reception, and explicit cross-tier interference constraints therefore remains insufficiently explored, leading to a highly non-convex mixed-integer design problem that requires scalable and structured solution strategies.

The main contributions of this work are summarized as follows:

1. We develop a unified IRS-assisted multi-layer aerial uplink system model that integrates UAV 3D deployment, IRS-enhanced composite channels, SIC-based uplink NOMA reception, and explicit shared-spectrum cross-tier interference constraints between HAP and UAV layers.
2. We formulate a joint uplink sum-rate maximization problem that rigorously incorporates practical system constraints, including UAV mobility limits, exclusive user association with NOMA cluster-size constraints, per-user and network-wide power budgets, minimum QOS requirements, unit-modulus IRS phase shifts, and cross-tier interference protection thresholds, ensuring feasible and reliable uplink operation.
3. To efficiently solve the resulting highly non-convex mixed-integer optimization problem, we propose a structured Block Coordinate Descent (BCD) framework that decomposes the original problem into tractable subproblems for UAV deployment, user association, uplink power control, and IRS phase optimization, each addressed using tailored optimization techniques with guaranteed convergence to a stationary point.
4. We provide a detailed computational complexity and convergence analysis of the proposed BCD-based framework, demonstrating that each subproblem can be solved with polynomial-time complexity and that the overall algorithm converges monotonically to a stationary point, making the approach suitable for practical large-scale IRS-assisted HAP–UAV uplink deployments.
5. Through extensive simulations, we demonstrate that the proposed design achieves substantial performance and reliability gains over conventional benchmarks, including significant improvements in uplink sum rate, outage probability, and rate reliability, while also providing system-level insights into the tradeoffs between UAV positioning, IRS deployment, and uplink NOMA clustering under dense user scheduling.

**Fig. 1:**
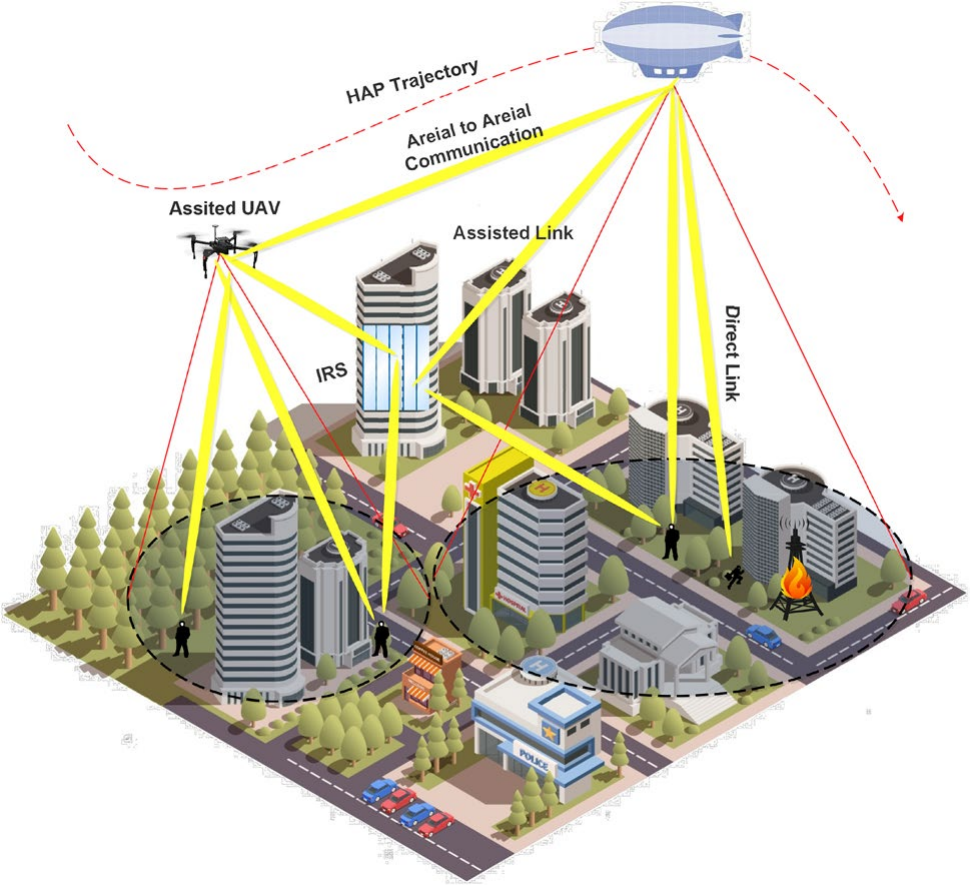
IRS-assisted hybrid HAP–UAV uplink NOMA communication network.

## System model

### Network architecture

We consider a dual-layer^30^ IRS-enabled HAP-assisted UAV-integrated uplink communication network designed to support single-antenna ground users in disaster and emergency scenarios. Let $\mathscr {K}=\{k \mid k=1,2,\ldots ,K\}$ denote the set of users, where each user transmits uplink data to exactly one aerial platform, either a HAP or an UAV, through direct air-to-ground links and, when available, IRS-assisted paths, as illustrated in Fig. 1. To accommodate dense user deployments, increasing traffic demand, and limited radio resources at the HAP, the UAV is deployed to complement the HAP by enhancing coverage and improving service flexibility. Accordingly, the users associated with the HAP and the UAV form two disjoint subsets, denoted by $\mathscr {K}_{\textrm{HAP}}\subseteq \mathscr {K}$ and $\mathscr {K}_{\textrm{UAV}}\subseteq \mathscr {K}$, respectively. Both the HAP and the UAV are equipped with multiple antennas and operate over a shared frequency spectrum. Uplink transmissions from users associated with the same aerial platform follow a NOMA principle, whereby multiple users simultaneously transmit over the same time–frequency resources and are separated at the receiver via SIC. To further enhance link quality and mitigate blockage effects caused by obstacles, such as high-rise buildings, a IRS comprising *M* passive reflecting elements is deployed to reconfigure the wireless propagation environment via intelligent adaptive phase adjustments. The HAP and UAV are interconnected via a dedicated inter-aerial link used exclusively for exchanging Channel State Information (CSI) and control signaling, without forwarding user data. This coordination enables effective interference management in the shared-spectrum uplink while preserving a purely uplink communication architecture. Note that the HAP’s position is assumed fixed during the considered scheduling interval. This design choice reflects the quasi-stationary nature of stratospheric HAPs, which operate on a significantly slower mobility timescale than agile low-altitude UAVs, thereby serving as stable wide-area anchor nodes rather than highly dynamic access points^31^.

### Channel model

Following^15^, perfect CSI is assumed to be available at both the UAV and the HAP, providing an upper bound on the achievable system performance^32^. The locations of ground users are obtained through dedicated control links and feedback mechanisms. The 3D Cartesian coordinates of ground user *k*, the UAV, the HAP, and the IRS are denoted by $\boldsymbol{L}_{k}=[x_k,y_k,h_k]^T$, $\boldsymbol{L}_{\text {UAV}}=[x_{\text {UAV}},y_{\text {UAV}},H_{\text {UAV}}]^T$, $\boldsymbol{L}_{\text {HAP}}=[x_{\text {HAP}},y_{\text {HAP}},H_{\text {HAP}}]^T$, and $\boldsymbol{L}_{\text {IRS}}=[x_{\text {IRS}},y_{\text {IRS}},H_{\text {IRS}}]^T$. The Euclidean distance between any two nodes *p* and *q* is given by $d_{p,q}=\Vert \boldsymbol{L}_p-\boldsymbol{L}_q\Vert$, where $d_{k,\text {UAV}}$, $d_{k,\text {HAP}}$, $d_{k,\text {IRS}}$, $d_{\text {IRS},\text {UAV}}$, and $d_{\text {IRS},\text {HAP}}$ denote the distances^8^ associated with the corresponding links. All large-scale channel gains are modeled in linear scale. For a link with distance *d*, the baseline Path Loss (PL) gain is expressed as:

1 $$\begin{aligned} \beta (d)=\beta _0\left( \frac{d_0}{d}\right) ^{\alpha }, \end{aligned}$$

where $d_0$ is the reference distance (typically 1 m), $\beta _0=\left( \frac{c}{4\pi f_c}\right) ^2$ denotes the free-space gain at $d_0$, $f_c$ is the carrier frequency, and $\alpha$ is the path-loss exponent. For Air-to-Ground (A2G) and Ground-to-Air (G2A) links, we consider probabilistic LoS and Non-Line-of-Sight (NLoS) propagation for the large-scale path loss. The LOS probability $p_{\text {LoS}}(d)$ follows the model in^15^, and the effective large-scale gain is given by^33^:

2 $$\begin{aligned} \bar{\beta }(d)=p_{\text {LoS}}(d)\beta _{\text {LoS}}(d)+\big (1-p_{\text {LoS}}(d)\big )\beta _{\text {NLoS}}(d), \end{aligned}$$

where $\beta _{\text {LoS}}(d)$ and $\beta _{\text {NLoS}}(d)$ follow the model in (1) with path-loss exponents $\alpha _{\text {LoS}}$ and $\alpha _{\text {NLoS}}$, respectively. Log-normal shadowing is incorporated via a multiplicative factor $S_{p,q}=10^{\frac{X_{p,q}}{10}},\quad X_{p,q}\sim \mathscr {N}(0,\sigma _{p,q}^2)$. The UAV and HAP are equipped with Uniform Linear Arrays (ULAs) of *N* antennas, while the IRS consists of *M* passive reflecting elements. Let $d_a$ denote the antenna spacing and $\lambda =c/f_c$ the carrier wavelength. The ULA steering vector of size *N* is defined as:

3 $$\begin{aligned} \boldsymbol{a}_N(\vartheta )= \left[ 1,e^{-j\frac{2\pi d_a}{\lambda }\sin (\vartheta )},\ldots , e^{-j(N-1)\frac{2\pi d_a}{\lambda }\sin (\vartheta )}\right] ^T, \end{aligned}$$

and the IRS steering vector $\boldsymbol{a}_M(\psi )$ is defined analogously. Small-scale fading is modeled as Rician fading, where the Rician factor $\kappa$ is selected according to the link type and propagation environment (i.e., LOS-dominant or NLOS-dominant), following^15^. For LOS-dominant links, $\kappa$ takes a large value, while for NLOS-dominant links, $\kappa$ approaches zero, reducing to Rayleigh fading. The LOS component of the Rician channel is modeled via array responses. For a generic channel, the fading matrix/vector is expressed as:

4 $$\begin{aligned} \textbf{G}= \sqrt{\frac{\kappa }{\kappa +1}}\textbf{G}_{\text {LoS}} + \sqrt{\frac{1}{\kappa +1}}\textbf{G}_{\text {NLoS}}, \end{aligned}$$

where $\kappa$ denotes the Rician factor. In (4), $\textbf{G}_{\text {LoS}}$ represents a deterministic component capturing the dominant propagation direction (e.g., via array responses), while $\textbf{G}_{\text {NLoS}}$ models diffuse scattering with i.i.d. $\mathscr{C}\mathscr{N}(0,1)$ entries. In the sequel, $\textbf{g}_{(\cdot )}$ denotes the corresponding Rician fading realizations for each channel. Accordingly, the uplink channels from user *k* to the UAV and HAP are given by:

5 $$\begin{aligned} \textbf{h}_{k,\text {UAV}}&=\sqrt{\bar{\beta }(d_{k,\text {UAV}})S_{k,\text {UAV}}}\,\textbf{g}_{k,\text {UAV}},\end{aligned}$$

6 $$\begin{aligned} \textbf{h}_{k,\text {HAP}}&=\sqrt{\bar{\beta }(d_{k,\text {HAP}})S_{k,\text {HAP}}}\,\textbf{g}_{k,\text {HAP}}. \end{aligned}$$

where $\textbf{g}_{k,\text {UAV}}, \textbf{g}_{k,\text {HAP}}\in \mathbb {C}^{N\times 1}$ follow the Rician fading model in (4). The user-to-IRS channel is modeled as:

7 $$\begin{aligned} \textbf{h}_{k,\text {IRS}}=\sqrt{\bar{\beta }(d_{k,\text {IRS}})S_{k,\text {IRS}}}\,\textbf{g}_{k,\text {IRS}}, \end{aligned}$$

with $\textbf{g}_{k,\text {IRS}}\in \mathbb {C}^{M\times 1}$ following the Rician fading model. The IRS-to-UAV and IRS-to-HAP channels are denoted by $\textbf{G}_{\text {IRS},\text {UAV}}\in \mathbb {C}^{N\times M}$ and $\textbf{G}_{\text {IRS},\text {HAP}}\in \mathbb {C}^{N\times M}$, respectively. Specifically, $\textbf{G}_{\text {IRS},r} = \sqrt{\bar{\beta }(d_{\text {IRS},r})S_{\text {IRS},r}}\,\textbf{g}_{\text {IRS},r}$ for $r\in \{\text {UAV},\text {HAP}\}$, where $\textbf{g}_{\text {IRS},r}\in \mathbb {C}^{N\times M}$ follows the Rician fading model in (4). For ideal passive IRS elements with unit amplitude reflection, the IRS reflection matrix is defined as:

8 $$\begin{aligned} {\boldsymbol{\Phi }}=\textrm{diag}\!\left( e^{j\varphi _1},e^{j\varphi _2},\ldots ,e^{j\varphi _M}\right) , \end{aligned}$$

where $\varphi _m\in [0,2\pi )$ denotes the phase shift applied by the *m*-th reflecting element. Finally, the effective uplink channels combining direct and IRS-assisted components are expressed as

9 $$\begin{aligned} \textbf{H}_{k,\text {UAV}}&= \textbf{h}_{k,\text {UAV}}+\textbf{G}_{\text {IRS},\text {UAV}}{\boldsymbol{\Phi }}\textbf{h}_{k,\text {IRS}},\end{aligned}$$

10 $$\begin{aligned} \textbf{H}_{k,\text {HAP}}&= \textbf{h}_{k,\text {HAP}}+\textbf{G}_{\text {IRS},\text {HAP}}{\boldsymbol{\Phi }}\textbf{h}_{k,\text {IRS}}. \end{aligned}$$

These effective channels serve as the basis for the received-signal model, SINR analysis, and the subsequent optimization framework.

### Communication model

Based on the channel model described in the previous subsection, we describe the uplink signal transmission and reception in the IRS-assisted HAP–UAV integrated network. Each user $k\in \mathscr {K}$ is associated with exactly one aerial platform, either the UAV or the HAP, as indicated by the binary variable $\delta _{k,r}\in \{0,1\}$, $r\in \{\text {UAV},\text {HAP}\}$, satisfying $\delta _{k,\text {UAV}}+\delta _{k,\text {HAP}}=1$.

Based on the association variables $\{\delta _{k,r}\}$, we define $\mathscr {K}_r \triangleq \{ k \in \mathscr {K} \mid \delta _{k,r}=1 \}, \quad r\in \{\text {UAV},\text {HAP}\}.$ Accordingly, $\mathscr {K}_{\text {UAV}}$ and $\mathscr {K}_{\text {HAP}}$ denote the sets of users associated with the UAV and the HAP, respectively. All users transmit simultaneously on the same frequency band, resulting in both intra-platform and cross-tier interference. Let $x_k$ denote the uplink symbol transmitted by user *k* with $\mathbb {E}[|x_k|^2]=1$ and transmit power $P_k=a_k P_k^{\max }$, where $a_k\in [0,1]$ is the power allocation coefficient and $P_k^{\max }$ is the maximum transmit power of user *k*. The received signal at the UAV can be written as:

11 $$\begin{aligned} \textbf{y}_{\text {UAV}} = {\sum }_{k\in \mathscr {K}_{\text {UAV}}} \sqrt{P_k}\,\textbf{H}_{k,\text {UAV}} x_k + {\sum }_{j\in \mathscr {K}_{\text {HAP}}} \sqrt{P_j}\,\textbf{H}_{j,\text {UAV}} x_j + \textbf{n}_{\text {UAV}}, \end{aligned}$$

where $\textbf{H}_{k,\text {UAV}}$ denotes the effective uplink channel from user *k* to the UAV and $\textbf{n}_{\text {UAV}}\sim \mathscr{C}\mathscr{N}(\textbf{0},\sigma _{\text {UAV}}^2\textbf{I})$ is the additive noise. The first summation represents the desired signals from UAV-associated users, while the second summation accounts for cross-tier interference from HAP-associated users. Similarly, the received signal at the HAP is expressed as:

12 $$\begin{aligned} \textbf{y}_{\text {HAP}} = {\sum }_{k\in \mathscr {K}_{\text {HAP}}} \sqrt{P_k}\,\textbf{H}_{k,\text {HAP}} x_k + {\sum }_{j\in \mathscr {K}_{\text {UAV}}} \sqrt{P_j}\,\textbf{H}_{j,\text {HAP}} x_j + \textbf{n}_{\text {HAP}}, \end{aligned}$$

where $\textbf{n}_{\text {HAP}}\sim \mathscr{C}\mathscr{N}(\textbf{0},\sigma _{\text {HAP}}^2\textbf{I})$. To exploit the multi-antenna capability of the UAV and the HAP, linear receive combining is applied. In particular, Maximum Ratio Combining (MRC) is adopted, yielding the combining vector for user *k* at receiver *r* as $\textbf{w}_{k,r}=\textbf{H}_{k,r}/\Vert \textbf{H}_{k,r}\Vert$. After applying MRC, the received signal for user *k* at receiver *r* becomes $y_{k,r} = \textbf{w}_{k,r}^H \textbf{y}_r.$ Uplink NOMA with SIC is employed independently at each aerial platform. For each receiver $r\in \{\text {UAV},\text {HAP}\}$, let $\pi _r(\cdot )$ denote the decoding order obtained by sorting users in $\mathscr {K}_r$ in descending order of their effective channel gains $\Vert \textbf{H}_{k,r}\Vert ^2$, which is used for uplink SIC. Users in $\mathscr {K}_r$ are ordered according to the adopted decoding order based on their effective channel gains $\Vert \textbf{H}_{k,r}\Vert ^2$, and SIC is applied such that, when decoding user *k*, signals of users with larger $\Vert \textbf{H}_{i,r}\Vert ^2$ have been decoded and canceled, while users with smaller $\Vert \textbf{H}_{i,r}\Vert ^2$ remain as interference. Signals transmitted by users associated with the other platform are treated as interference without decoding. The adopted power control and association are assumed to satisfy SIC feasibility at each receiver; explicit SIC decodability constraints can be incorporated if needed. Accordingly, the post-combining SINR for user *k* associated with receiver *r* under MRC is given by:

13 $$\begin{aligned} \textrm{SINR}_{k,r} = \frac{P_k \Vert \textbf{H}_{k,r}\Vert ^2}{ \sum \limits _{\begin{array}{c} i\in \mathscr {K}_r \\ \Vert \textbf{H}_{i,r}\Vert ^2 < \Vert \textbf{H}_{k,r}\Vert ^2 \end{array}} P_i \frac{|\textbf{H}_{k,r}^H\textbf{H}_{i,r}|^2}{\Vert \textbf{H}_{k,r}\Vert ^2} + \sum \limits _{j\in \mathscr {K}_{\bar{r}}} P_j \frac{|\textbf{H}_{k,r}^H\textbf{H}_{j,r}|^2}{\Vert \textbf{H}_{k,r}\Vert ^2} + \sigma _r^2}, \end{aligned}$$

where $\bar{r}$ represents the complementary platform ($\bar{r} = \text {HAP}$ if $r = \text {UAV}$, and vice versa), and $\sigma _r^2$ is the noise variance. The first term in the denominator captures residual intra-platform interference from weaker users under NOMA- SIC, while the second term accounts for cross-tier interference. Finally, the achievable uplink rate of user *k* is given by $R_{k,r}=B\log _2\big (1+\textrm{SINR}_{k,r}\big )$, which serves as the basis for subsequent performance analysis and optimization.

### Joint optimization problem

Based on the communication model described previously, we jointly optimize the UAV 3D deployment, user association, uplink power allocation, and IRS phase shifts to maximize the uplink sum rate under practical QOS, NOMA, interference, and mobility constraints. Let $\textbf{L}_{\text {UAV}}=[x_{\text {UAV}},y_{\text {UAV}},H_{\text {UAV}}]^T$ denote the UAV position, $\delta _{k,r}\in \{0,1\}$ the association of user *k* to receiver $r\in \{\text {UAV},\text {HAP}\}$, $a_k\in [0,1]$ the power coefficient with $P_k=a_kP_k^{\max }$, and $\boldsymbol{\Phi }=\textrm{diag}(e^{j\varphi _1},\ldots ,e^{j\varphi _M})$ the IRS reflection matrix. The achievable rate of user *k* is $R_{k,r}=B\log _2(1+\textrm{SINR}_{k,r})$, where $\textrm{SINR}_{k,r}$ follows the NOMA- SIC uplink model defined in (13) and depends on $\textbf{L}_{\text {UAV}}$, $\{\delta _{k,r}\}$, $\{a_k\}$, and $\boldsymbol{\Phi }$ through the effective channels in (9)–(10).

The joint optimization is formulated as:

14a $$\begin{aligned} \max _{\textbf{L}_{\text {UAV}},\,\{\delta _{k,r}\},\,\{a_k\},\,{\boldsymbol{\Phi }}} \quad&{\sum }_{k\in \mathscr {K}}{{\sum }}_{r\in \{\text {UAV},\text {HAP}\}} \delta _{k,r}\, R_{k,r} \end{aligned}$$

14b $$\begin{aligned} \text {s.t.}\quad&\delta _{k,r}\in \{0,1\},\quad \forall k\in \mathscr {K},\, r\in \{\text {UAV},\text {HAP}\}, \end{aligned}$$

14c $$\begin{aligned}&\delta _{k,\text {UAV}}+\delta _{k,\text {HAP}}=1,\quad \forall k\in \mathscr {K}, \end{aligned}$$

14d $$\begin{aligned}&0\le a_k \le 1,\quad \forall k\in \mathscr {K}, \end{aligned}$$

14e $$\begin{aligned}&{{\sum }}_{k\in \mathscr {K}} a_k P_k^{\max } \le P_{\text {total}}^{\max }, \end{aligned}$$

14f $$\begin{aligned}&{{\sum }}_{k\in \mathscr {K}} \delta _{k,r} \le U_r,\quad \forall r\in \{\text {UAV},\text {HAP}\}, \end{aligned}$$

14g $$\begin{aligned}&\delta _{k,r}\,\textrm{SINR}_{k,r} \ge \delta _{k,r}\,\gamma _{k}^{\min },\quad \forall k\in \mathscr {K},\, r\in \{\text {UAV},\text {HAP}\}, \end{aligned}$$

14h $$\begin{aligned}&{{\sum }}_{k\in \mathscr {K}} \delta _{k,\text {HAP}} P_k \Vert \textbf{H}_{k,\text {UAV}}\Vert ^2 \le I_{\text {UAV}}^{\max }, \end{aligned}$$

14i $$\begin{aligned}&{{\sum }}_{k\in \mathscr {K}} \delta _{k,\text {UAV}} P_k \Vert \textbf{H}_{k,\text {HAP}}\Vert ^2 \le I_{\text {HAP}}^{\max }, \end{aligned}$$

14j $$\begin{aligned}&|[{\boldsymbol{\Phi }}]_{m,m}| = 1,\quad \forall m\in \{1,\ldots ,M\}, \end{aligned}$$

14k $$\begin{aligned}&\varphi _m \in [0, 2\pi ),\quad \forall m\in \{1,\ldots ,M\}, \end{aligned}$$

14l $$\begin{aligned}&x_{\min } \le x_{\text {UAV}} \le x_{\max }, \end{aligned}$$

14m $$\begin{aligned}&y_{\min } \le y_{\text {UAV}} \le y_{\max }, \end{aligned}$$

14n $$\begin{aligned}&H_{\min } \le H_{\text {UAV}} \le H_{\max }. \end{aligned}$$

Constraint (14e) imposes a network-wide uplink transmit power budget (e.g., reflecting emergency-operation energy limitations), while the individual power constraints are enforced through $0\le a_k\le 1$. In (14f), $U_r$ limits the number of simultaneously multiplexed users (i.e., the NOMA cluster size) at receiver *r* to control SIC complexity and error propagation. Constraint (14g) enforces per-user QOS requirements via minimum SINR thresholds $\gamma _k^{\min }$, where the multiplication by $\delta _{k,r}$ ensures the constraint applies only when user *k* is associated with receiver *r*. Constraints (14h)–(14i) bound the total cross-tier interference using the effective channel power gains $\Vert \textbf{H}_{k,r}\Vert ^2$, which provide a conservative surrogate under MRC, where $I_{\text {UAV}}^{\max }$ and $I_{\text {HAP}}^{\max }$ denote interference budgets at the UAV and HAP, respectively. Constraints (14j) and (14k) capture the passive IRS phase shift constraints with unit-modulus reflection coefficients. Constraints (14l)–(14n) restrict the UAV position within a feasible 3D flight region.

#### Remark 0.1

Problem (14) is a mixed-integer non-linear program^34^ (Mixed-Integer Non-Linear Program (MINLP)). The non-convexity arises from: (i) the coupled SINR expressions under NOMA- SIC, which involve fractional terms and combinatorial association; (ii) the unit-modulus IRS phase constraints; (iii) the highly non-linear dependence of channel gains on UAV position through (9)–(10); and (iv) the binary association variables. Thus, globally optimal solutions are generally intractable, motivating the use of efficient iterative or decomposition-based approaches.

## Proposed solution approach and BCD-IRS-HU framework

To address the highly non-convex and computationally challenging MINLP formulated in (14), we propose a Block Coordinate Descent (BCD)-based iterative optimization framework^15,35^. The BCD method decomposes the joint optimization problem into a series of more tractable subproblems by cyclically optimizing one block of variables while fixing the others. This approach effectively decouples the interdependent variables and enables the application of tailored optimization techniques to each subproblem. Specifically, we partition the optimization variables into four blocks: (1) UAV deployment $\textbf{L}_{\text {UAV}}$, (2) user association $\{\delta _{k,r}\}$, (3) power allocation $\{a_k\}$, and (4) IRS phase shifts ${\boldsymbol{\Phi }}$. The proposed BCD-IRS-HU framework then iteratively solves the following subproblems until convergence:

### UAV deployment subproblem

With fixed user association $\{\delta _{k,r}\}$, power allocation coefficients $\{a_k\}$, and IRS phase shift matrix ${\boldsymbol{\Phi }}$, the UAV deployment subproblem reduces to optimizing the 3D coordinates of the UAV, $\textbf{L}_{\text {UAV}} = [x_{\text {UAV}}, y_{\text {UAV}}, H_{\text {UAV}}]^T$, to maximize the sum rate of UAV-associated users while satisfying mobility constraints. Note that the rates of HAP-associated users remain constant with respect to $\textbf{L}_{\text {UAV}}$, as their channels to the HAP are independent of the UAV position. However, the UAV position affects both the desired signal strength for UAV-associated users and the cross-tier interference from HAP-associated users to the UAV.

The UAV deployment subproblem is mathematically formulated as:

15a $$\begin{aligned} \max _{\textbf{L}_{\text {UAV}}} \quad&f(\textbf{L}_{\text {UAV}}) = {\sum }_{k \in \mathscr {K}_{\text {UAV}}} B \log _2\left( 1 + \textrm{SINR}_{k,\text {UAV}}(\textbf{L}_{\text {UAV}})\right) \end{aligned}$$

15b $$\begin{aligned} \text {s.t.} \quad&I_{\text {UAV}}(\textbf{L}_{\text {UAV}}) = {\sum }_{j \in \mathscr {K}_{\text {HAP}}} P_j \Vert \textbf{H}_{j,\text {UAV}}(\textbf{L}_{\text {UAV}})\Vert ^2 \le I_{\text {UAV}}^{\max }, \end{aligned}$$

15c $$\begin{aligned}&\textrm{SINR}_{k,\text {UAV}}(\textbf{L}_{\text {UAV}}) \ge \gamma _k^{\min }, \quad \forall k \in \mathscr {K}_{\text {UAV}}, \end{aligned}$$

15d $$\begin{aligned}&x_{\min } \le x_{\text {UAV}} \le x_{\max }, \end{aligned}$$

15e $$\begin{aligned}&y_{\min } \le y_{\text {UAV}} \le y_{\max }, \end{aligned}$$

15f $$\begin{aligned}&H_{\min } \le H_{\text {UAV}} \le H_{\max }. \end{aligned}$$

In (15), the objective function $f(\textbf{L}_{\text {UAV}})$ represents the sum rate of all UAV-associated users, where $\textrm{SINR}_{k,\text {UAV}}(\textbf{L}_{\text {UAV}})$ follows the expression in (13) with $r = \text {UAV}$. The effective channel $\textbf{H}_{k,\text {UAV}}(\textbf{L}_{\text {UAV}})$ depends on the UAV position through both the direct path and the IRS-assisted path, as defined in (9). Constraint (15b) ensures that the total cross-tier interference power from HAP-associated users to the UAV remains below the threshold $I_{\text {UAV}}^{\max }$. Constraints (15c) guarantee the minimum SINR requirements for UAV-associated users. Constraints (15d)–(15f) bound the UAV’s 3D position within the feasible flight region.

The optimization problem (15) is non-convex due to the complicated dependence of the SINR expressions on $\textbf{L}_{\text {UAV}}$ through the composite channel gains and interference terms. To address this challenge, we employ a Successive Convex Approximation (Successive Convex Approximation (SCA)) approach with a trust region. At each iteration, we linearize the objective function and constraints around the current UAV position and solve a convex approximation within a trust region.

Let $\textbf{L}_{\text {UAV}}^{(t)} = [x^{(t)}, y^{(t)}, H^{(t)}]^T$ denote the UAV position at iteration *t*. We compute first-order Taylor approximations of the objective and constraint functions:

16 $$\begin{aligned} \tilde{f}(\textbf{L}_{\text {UAV}}; \textbf{L}_{\text {UAV}}^{(t)})&= f(\textbf{L}_{\text {UAV}}^{(t)}) + \nabla f(\textbf{L}_{\text {UAV}}^{(t)})^T (\textbf{L}_{\text {UAV}} - \textbf{L}_{\text {UAV}}^{(t)}), \end{aligned}$$

17 $$\begin{aligned} \tilde{I}_{\text {UAV}}(\textbf{L}_{\text {UAV}}; \textbf{L}_{\text {UAV}}^{(t)})&= I_{\text {UAV}}(\textbf{L}_{\text {UAV}}^{(t)}) + \nabla I_{\text {UAV}}(\textbf{L}_{\text {UAV}}^{(t)})^T (\textbf{L}_{\text {UAV}} - \textbf{L}_{\text {UAV}}^{(t)}), \end{aligned}$$

18 $$\begin{aligned} \widetilde{\textrm{SINR}}_{k,\text {UAV}}(\textbf{L}_{\text {UAV}}; \textbf{L}_{\text {UAV}}^{(t)})&= \textrm{SINR}_{k,\text {UAV}}(\textbf{L}_{\text {UAV}}^{(t)}) + \nabla \textrm{SINR}_{k,\text {UAV}}(\textbf{L}_{\text {UAV}}^{(t)})^T (\textbf{L}_{\text {UAV}} - \textbf{L}_{\text {UAV}}^{(t)}), \quad \forall k \in \mathscr {K}_{\text {UAV}}. \end{aligned}$$

The gradients $\nabla f$, $\nabla I_{\text {UAV}}$, and $\nabla \textrm{SINR}_{k,\text {UAV}}$ are computed using the chain rule, taking into account the dependence of channel gains on distances and angles, which in turn depend on $\textbf{L}_{\text {UAV}}$. For implementation, these gradients can be obtained analytically or numerically, e.g., via finite differences. The approximated convex subproblem at iteration *t* is then formulated as:

19a $$\begin{aligned} \max _{\textbf{L}_{\text {UAV}}} \quad&\tilde{f}(\textbf{L}_{\text {UAV}}; \textbf{L}_{\text {UAV}}^{(t)}) \end{aligned}$$

19b $$\begin{aligned} \text {s.t.} \quad&\tilde{I}_{\text {UAV}}(\textbf{L}_{\text {UAV}}; \textbf{L}_{\text {UAV}}^{(t)}) \le I_{\text {UAV}}^{\max }, \end{aligned}$$

19c $$\begin{aligned}&\widetilde{\textrm{SINR}}_{k,\text {UAV}}(\textbf{L}_{\text {UAV}}; \textbf{L}_{\text {UAV}}^{(t)}) \ge \gamma _k^{\min }, \quad \forall k \in \mathscr {K}_{\text {UAV}}, \end{aligned}$$

19d $$\begin{aligned}&x_{\min } \le x_{\text {UAV}} \le x_{\max }, \quad y_{\min } \le y_{\text {UAV}} \le y_{\max }, \quad H_{\min } \le H_{\text {UAV}} \le H_{\max }, \end{aligned}$$

19e $$\begin{aligned}&\Vert \textbf{L}_{\text {UAV}} - \textbf{L}_{\text {UAV}}^{(t)}\Vert _\infty \le \Delta ^{(t)}, \end{aligned}$$

where $\Delta ^{(t)}> 0$ is the trust region radius at iteration *t*. Constraint (19e) ensures that the new solution remains within a neighborhood of the current point, maintaining the validity of the linear approximations. Problem (19) is a linear program (LP) that can be solved efficiently using standard convex optimization solvers. The trust region radius $\Delta ^{(t)}$ is adaptively updated based on the ratio of actual to predicted improvement:

$$\rho ^{(t)} = \frac{f(\textbf{L}_{\text {UAV}}^{(t+1)}) - f(\textbf{L}_{\text {UAV}}^{(t)})}{\tilde{f}(\textbf{L}_{\text {UAV}}^{(t+1)}; \textbf{L}_{\text {UAV}}^{(t)}) - \tilde{f}(\textbf{L}_{\text {UAV}}^{(t)}; \textbf{L}_{\text {UAV}}^{(t)})},$$

where $\textbf{L}_{\text {UAV}}^{(t+1)}$ is the solution of (19). If $\rho ^{(t)}$ is close to 1, the approximation is accurate, and we increase $\Delta ^{(t)}$; if $\rho ^{(t)}$ is small or negative, we decrease $\Delta ^{(t)}$ to improve the approximation quality. The SCA algorithm for UAV deployment is summarized in Algorithm 1. The algorithm terminates when the change in the objective function or the UAV position falls below a predefined threshold $\epsilon$, indicating convergence to a locally optimal solution.

**Algorithm 1:**
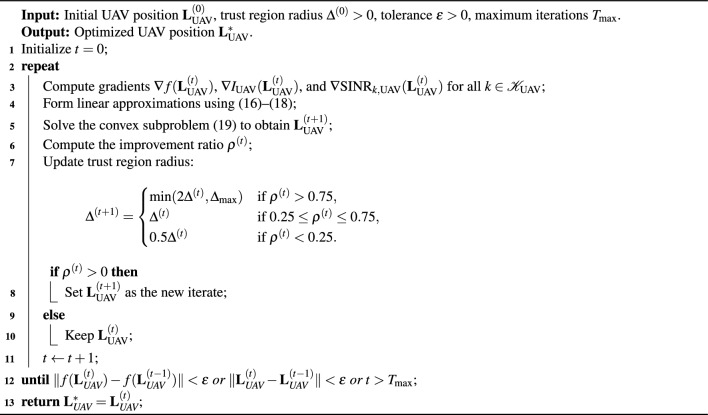
UAV deployment via Successive Convex Approximation.

The proposed SCA-based approach guarantees convergence to a locally optimal solution of the UAV deployment subproblem while maintaining computational efficiency through convex approximations. The optimized UAV position enhances the effective channels for UAV-associated users while controlling cross-tier interference, thereby improving the overall network performance.

### User association subproblem

Given fixed UAV position $\textbf{L}_{\text {UAV}}$, power allocation coefficients $\{a_k\}$, and IRS phase shift matrix ${\boldsymbol{\Phi }}$, the user association subproblem aims to assign each user $k \in \mathscr {K}$ to either the UAV or the HAP by optimizing the binary variables $\{\delta _{k,r}\}$, where $r \in \{\text {UAV}, \text {HAP}\}$. The objective is to maximize the sum rate while satisfying the exclusive association and cluster size constraints. Mathematically, the subproblem is formulated as:

20a $$\begin{aligned} \max _{\{\delta _{k,r}\}} \quad&{\sum }_{k \in \mathscr {K}} {\sum }_{r \in \{\text {UAV},\text {HAP}\}} \delta _{k,r} R_{k,r} \end{aligned}$$

20b $$\begin{aligned} \text {s.t.} \quad&\delta _{k,r} \in \{0,1\}, \quad \forall k \in \mathscr {K}, \; r \in \{\text {UAV},\text {HAP}\}, \end{aligned}$$

20c $$\begin{aligned}&\delta _{k,\text {UAV}} + \delta _{k,\text {HAP}} = 1, \quad \forall k \in \mathscr {K}, \end{aligned}$$

20d $$\begin{aligned}&{\sum }_{k \in \mathscr {K}} \delta _{k,r} \le U_r, \quad \forall r \in \{\text {UAV},\text {HAP}\}, \end{aligned}$$

where $R_{k,r} = B \log _2(1 + \textrm{SINR}_{k,r})$ is the achievable rate of user *k* when associated with receiver *r*, and $\textrm{SINR}_{k,r}$ is given by (13). The main challenge in solving (20) directly is the strong coupling among the association variables through the interference terms in the SINR expressions. Specifically, intra-platform interference experienced by a user depends on which other users are assigned to the same receiver. In contrast, cross-tier interference depends on the assignment to the opposite receiver. To efficiently tackle this combinatorial problem, we propose an iterative algorithm based on the concept of *fixed interference approximation*. The key idea is to decouple the association decisions by approximating the interference terms using the association pattern from the previous iteration. At each iteration, we compute average interference levels for each platform, then solve a decoupled assignment problem that maximizes the sum rate under the approximation. Let $\mathscr {K}_r^{(t)}$ denote the set of users associated with receiver *r* at iteration *t*. We define the following average interference measures:

- **Average intra-platform interference** at receiver *r*:
  21 $$\begin{aligned} \bar{I}_{\text {intra},r}^{(t)} = \frac{1}{|\mathscr {K}_r^{(t)}|} {\sum }_{k \in \mathscr {K}_r^{(t)}} {\sum }_{\begin{array}{c} i \in \mathscr {K}_r^{(t)} \\ i \ne k, \; \Vert \textbf{H}_{i,r}\Vert ^2 < \Vert \textbf{H}_{k,r}\Vert ^2 \end{array}} P_i \frac{|\textbf{H}_{k,r}^H \textbf{H}_{i,r}|^2}{\Vert \textbf{H}_{k,r}\Vert ^2}. \end{aligned}$$
- **Average cross-tier interference** from the opposite platform $r'$ to receiver *r*:
  22 $$\begin{aligned} \bar{I}_{\text {inter},r}^{(t)} = \frac{1}{|\mathscr {K}_r^{(t)}|} {\sum }_{k \in \mathscr {K}_r^{(t)}} {\sum }_{j \in \mathscr {K}_{r'}^{(t)}} P_j \frac{|\textbf{H}_{k,r}^H \textbf{H}_{j,r}|^2}{\Vert \textbf{H}_{k,r}\Vert ^2}, \quad r' \ne r. \end{aligned}$$

These averages are computed using the channel gains and transmit powers, which are fixed from the outer BCD loop. With these, we approximate the SINR for user *k* on platform *r* as:

23 $$\begin{aligned} \widetilde{\textrm{SINR}}_{k,r}^{(t)} = \frac{P_k \Vert \textbf{H}_{k,r}\Vert ^2}{\bar{I}_{\text {intra},r}^{(t)} + \bar{I}_{\text {inter},r}^{(t)} + \sigma _r^2}, \end{aligned}$$

and the corresponding approximate rate as $\widetilde{R}_{k,r}^{(t)} = B \log _2(1 + \widetilde{\textrm{SINR}}_{k,r}^{(t)})$. Under this approximation, the objective function becomes linear in the association variables:

24 $$\begin{aligned} {\sum }_{k \in \mathscr {K}} {\sum }_{r} \delta _{k,r} \widetilde{R}_{k,r}^{(t)}. \end{aligned}$$

The decoupled assignment problem at iteration *t* is then:

25a $$\begin{aligned} \max _{\{\delta _{k,r}\}} \quad&{\sum }_{k \in \mathscr {K}} {\sum }_{r} \delta _{k,r} \widetilde{R}_{k,r}^{(t)} \end{aligned}$$

25b $$\begin{aligned} \text {s.t.} \quad&(20b), \; (20c), \; (20d). \end{aligned}$$

Problem (25) is a binary linear program with two knapsack constraints. Thanks to the exclusive association constraint, it can be solved optimally via a simple sorting procedure. Define the rate difference for user *k* as:

26 $$\begin{aligned} d_k^{(t)} = \widetilde{R}_{k,\text {UAV}}^{(t)} - \widetilde{R}_{k,\text {HAP}}^{(t)}. \end{aligned}$$

Intuitively, $d_k^{(t)}$ measures the preference for associating user *k* with the UAV versus the HAP. Let the users be sorted in descending order of $d_k^{(t)}$, so that $d_{[1]}^{(t)} \ge d_{[2]}^{(t)} \ge \cdots \ge d_{[K]}^{(t)}$, where [*i*] denotes the index of the *i*-th user in the sorted list. The capacity constraints (20d) impose that at most $U_{\text {UAV}}$ users can be assigned to the UAV and at most $U_{\text {HAP}}$ to the HAP. Since every user must be assigned, we also require that the number of users assigned to the HAP, $K - n_{\text {UAV}}$, does not exceed $U_{\text {HAP}}$, i.e., $n_{\text {UAV}} \ge K - U_{\text {HAP}}$. Therefore, the number of users assigned to the UAV must satisfy:

27 $$\begin{aligned} L \le n_{\text {UAV}} \le U_{\text {UAV}}, \quad \text {where } L = \max (0, K - U_{\text {HAP}}). \end{aligned}$$

The optimal $n_{\text {UAV}}^{*}$ is chosen to maximize the sum of the top $n_{\text {UAV}}$ rate differences. Because the sequence $d_{[i]}^{(t)}$ is sorted descending, the sum of the first *n* terms is non-decreasing as long as $d_{[n]}^{(t)}> 0$. Hence, the optimal solution is:

28 $$\begin{aligned} n_{\text {UAV}}^{*} = {\left\{ \begin{array}{ll} \min \!\big (U_{\text {UAV}}, \, M^{(t)}\big ), & \text {if } \min \!\big (U_{\text {UAV}}, M^{(t)}\big ) \ge L, \\ L, & \text {otherwise}, \end{array}\right. } \end{aligned}$$

where $M^{(t)}$ is the number of users with $d_k^{(t)}> 0$. The association is then:

$$\delta _{[i],\text {UAV}} = {\left\{ \begin{array}{ll} 1, & \text {for } i = 1, \dots , n_{\text {UAV}}^{*},\\ 0, & \text {for } i = n_{\text {UAV}}^{*}+1, \dots , K, \end{array}\right. }$$

and $\delta _{[i],\text {HAP}} = 1 - \delta _{[i],\text {UAV}}$. The overall iterative algorithm for the user association subproblem is summarized in Algorithm 2. The algorithm terminates when the association pattern converges or a maximum number of iterations is reached. In practice, we observe rapid convergence within a few iterations.

**Algorithm 2:**
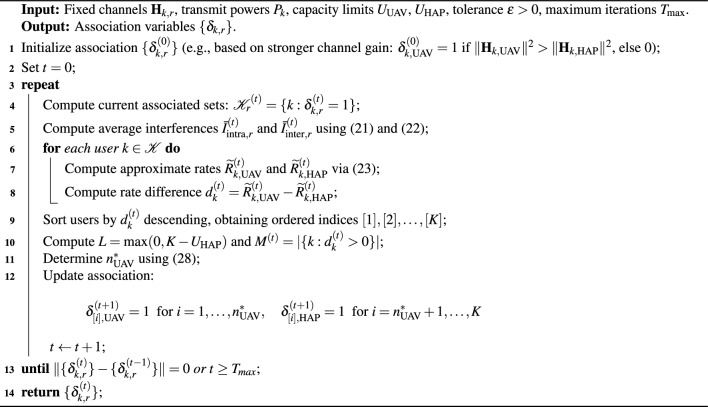
User Association via Fixed Interference Approximation.

The proposed algorithm efficiently solves the user association subproblem by leveraging fixed interference approximations and a sorting-based optimal assignment. Its low computational complexity makes it suitable for integration into the overall BCD framework. Moreover, the approximation becomes increasingly accurate as the association pattern stabilizes, ensuring that the algorithm converges to a high-quality solution.

### Power allocation subproblem

With fixed UAV deployment $\textbf{L}_{\text {UAV}}$, user association $\{\delta _{k,r}\}$, and IRS phase shift matrix $\boldsymbol{\Phi }$, the power allocation subproblem optimizes the power control coefficients $\{a_k\}$ to maximize the sum rate under per-user power limits, total power budget, QOS requirements, and interference constraints. The subproblem is formulated as:

29a $$\begin{aligned} \max _{\{a_k\}} \quad&{\sum }_{k \in \mathscr {K}} {\sum }_{r \in \{\text {UAV},\text {HAP}\}} \delta _{k,r} B \log _2\left( 1 + \textrm{SINR}_{k,r}\right) \end{aligned}$$

29b $$\begin{aligned} \text {s.t.} \quad&0 \le a_k \le 1, \quad \forall k \in \mathscr {K}, \end{aligned}$$

29c $$\begin{aligned}&{\sum }_{k \in \mathscr {K}} a_k P_k^{\max } \le P_{\text {total}}^{\max }, \end{aligned}$$

29d $$\begin{aligned}&\delta _{k,r} \, \textrm{SINR}_{k,r} \ge \delta _{k,r} \, \gamma _k^{\min }, \quad \forall k \in \mathscr {K}, \; r \in \{\text {UAV},\text {HAP}\}, \end{aligned}$$

29e $$\begin{aligned}&{\sum }_{k \in \mathscr {K}} \delta _{k,\text {HAP}} a_k P_k^{\max } \Vert \textbf{H}_{k,\text {UAV}}\Vert ^2 \le I_{\text {UAV}}^{\max }, \end{aligned}$$

29f $$\begin{aligned}&{\sum }_{k \in \mathscr {K}} \delta _{k,\text {UAV}} a_k P_k^{\max } \Vert \textbf{H}_{k,\text {HAP}}\Vert ^2 \le I_{\text {HAP}}^{\max }, \end{aligned}$$

where $\textrm{SINR}_{k,r}$ is given by (13). The main difficulty in solving (29) stems from the non-convex SINR expressions in both the objective and the QOS constraints. To tackle this, we employ the *quadratic transform* technique from fractional programming, which decouples the fractional terms into more tractable forms. We first rewrite the $\textrm{SINR}_{k,r}$ for user *k* associated with *r* as:

30 $$\begin{aligned} \textrm{SINR}_{k,r} = \frac{P_k |\textbf{H}_{k,r}^H \textbf{H}_{k,r}|}{{\sum }_{\begin{array}{c} i \in \mathscr {K}_r \\ i \ne k, \Vert \textbf{H}_{i,r}\Vert ^2 < \Vert \textbf{H}_{k,r}\Vert ^2 \end{array}} P_i \frac{|\textbf{H}_{k,r}^H \textbf{H}_{i,r}|^2}{\Vert \textbf{H}_{k,r}\Vert ^2} + {\sum }_{j \in \mathscr {K}_{\bar{r}}} P_j \frac{|\textbf{H}_{k,r}^H \textbf{H}_{j,r}|^2}{\Vert \textbf{H}_{k,r}\Vert ^2} + \sigma _r^2}. \end{aligned}$$

Note that $P_k = a_k P_k^{\max }$ and $|\textbf{H}_{k,r}^H \textbf{H}_{k,r}| = \Vert \textbf{H}_{k,r}\Vert ^4$. Define the following terms for compactness:

31 $$\begin{aligned} s_{k,r}&= \Vert \textbf{H}_{k,r}\Vert ^4, \quad \text {(signal power coefficient)} \end{aligned}$$

32 $$\begin{aligned} I_{i,k,r}^{\text {intra}}&= \frac{|\textbf{H}_{k,r}^H \textbf{H}_{i,r}|^2}{\Vert \textbf{H}_{k,r}\Vert ^2}, \quad i \in \mathscr {K}_r, \; i \ne k, \; \Vert \textbf{H}_{i,r}\Vert ^2 < \Vert \textbf{H}_{k,r}\Vert ^2, \end{aligned}$$

33 $$\begin{aligned} I_{j,k,r}^{\text {inter}}&= \frac{|\textbf{H}_{k,r}^H \textbf{H}_{j,r}|^2}{\Vert \textbf{H}_{k,r}\Vert ^2}, \quad j \in \mathscr {K}_{\bar{r}}. \end{aligned}$$

These coefficients are constants given the fixed channels. Then, the SINR becomes:

34 $$\begin{aligned} \textrm{SINR}_{k,r} = \frac{a_k P_k^{\max } s_{k,r}}{{\sum }_{i} a_i P_i^{\max } I_{i,k,r}^{\text {intra}} + {\sum }_{j} a_j P_j^{\max } I_{j,k,r}^{\text {inter}} + \sigma _r^2}. \end{aligned}$$

The quadratic transform introduces auxiliary variables $\{\beta _{k,r}\}$ for each SINR term. For a fractional term of the form $\frac{A(x)}{B(x)}$, the quadratic transform yields a surrogate function: $2\sqrt{A(x)} \beta - \beta ^2 B(x).$ Applying this to each SINR, we define the following transformed function for the objective:

35 $$\begin{aligned} \tilde{R}_{k,r}(a_k, \beta _{k,r}) = B \log _2\left( 1 + 2\beta _{k,r} \sqrt{a_k P_k^{\max } s_{k,r}} - \beta _{k,r}^2 \left( {\sum }_{i} a_i P_i^{\max } I_{i,k,r}^{\text {intra}} + {\sum }_{j} a_j P_j^{\max } I_{j,k,r}^{\text {inter}} + \sigma _r^2 \right) \right) . \end{aligned}$$

Similarly, for the QOS constraints $\delta _{k,r} {SINR}_{k,r} \ge \delta _{k,r} \gamma _k^{\min }$, we introduce auxiliary variables $\{\alpha _{k,r}\}$ and rewrite each constraint as:

36 $$\begin{aligned} 2\alpha _{k,r} \sqrt{a_k P_k^{\max } s_{k,r}} - \alpha _{k,r}^2 \left( {\sum }_{i} a_i P_i^{\max } I_{i,k,r}^{\text {intra}} + {\sum }_{j} a_j P_j^{\max } I_{j,k,r}^{\text {inter}} + \sigma _r^2 \right) \ge \gamma _k^{\min }. \end{aligned}$$

Given fixed $\beta _{k,r}$ and $\alpha _{k,r}$, both the objective and QOS constraints become concave in $\{a_k\}$ because the square root term is concave and the quadratic terms are linear. The interference constraints (29e)–(29f) and power constraints (29b)–(29c) are linear in $\{a_k\}$. Thus, we obtain a convex optimization problem in $\{a_k\}$ for fixed auxiliary variables. The auxiliary variables are updated optimally for fixed $\{a_k\}$ as:

37 $$\begin{aligned} \beta _{k,r}&= \frac{\sqrt{a_k P_k^{\max } s_{k,r}}}{{\sum }_{i} a_i P_i^{\max } I_{i,k,r}^{\text {intra}} + {\sum }_{j} a_j P_j^{\max } I_{j,k,r}^{\text {inter}} + \sigma _r^2}, \end{aligned}$$

38 $$\begin{aligned} \alpha _{k,r}&= \frac{\sqrt{a_k P_k^{\max } s_{k,r}}}{{\sum }_{i} a_i P_i^{\max } I_{i,k,r}^{\text {intra}} + {\sum }_{j} a_j P_j^{\max } I_{j,k,r}^{\text {inter}} + \sigma _r^2}. \end{aligned}$$

We alternate between optimizing $\{a_k\}$ and updating the auxiliary variables until convergence. The convex subproblem for $\{a_k\}$ at each iteration is:

39a $$\begin{aligned} \max _{\{a_k\}} \quad&{\sum }_{k \in \mathscr {K}} {\sum }_{r} \delta _{k,r} \tilde{R}_{k,r}(a_k, \beta _{k,r}) \end{aligned}$$

39b $$\begin{aligned} \text {s.t.} \quad&0 \le a_k \le 1, \quad \forall k \in \mathscr {K}, \end{aligned}$$

39c $$\begin{aligned}&{\sum }_{k \in \mathscr {K}} a_k P_k^{\max } \le P_{\text {total}}^{\max }, \end{aligned}$$

39d $$\begin{aligned}&2\alpha _{k,r} \sqrt{a_k P_k^{\max } s_{k,r}} - \alpha _{k,r}^2 \left( {\sum }_{i} a_i P_i^{\max } I_{i,k,r}^{\text {intra}} + {\sum }_{j} a_j P_j^{\max } I_{j,k,r}^{\text {inter}} + \sigma _r^2 \right) \ge \gamma _k^{\min }, \nonumber \\&\hspace{12em} \forall k \in \mathscr {K}, \; r \in \{\text {UAV},\text {HAP}\}, \end{aligned}$$

39e $$\begin{aligned}&{\sum }_{k \in \mathscr {K}} \delta _{k,\text {HAP}} a_k P_k^{\max } \Vert \textbf{H}_{k,\text {UAV}}\Vert ^2 \le I_{\text {UAV}}^{\max }, \end{aligned}$$

39f $$\begin{aligned}&{\sum }_{k \in \mathscr {K}} \delta _{k,\text {UAV}} a_k P_k^{\max } \Vert \textbf{H}_{k,\text {HAP}}\Vert ^2 \le I_{\text {HAP}}^{\max }. \end{aligned}$$

This problem is a convex optimization problem because:

- The objective (39a) is a sum of logarithms of concave functions (inside the log is concave in $a_k$), which is concave.
- All constraints are linear or convex: (39b) and (39c) are linear, (39d) is convex (a concave function minus a linear term), and (39e)–(39f) are linear.

Thus, it can be efficiently solved using interior-point methods. The overall power-allocation algorithm is summarized in Algorithm 3.

**Algorithm 3:**
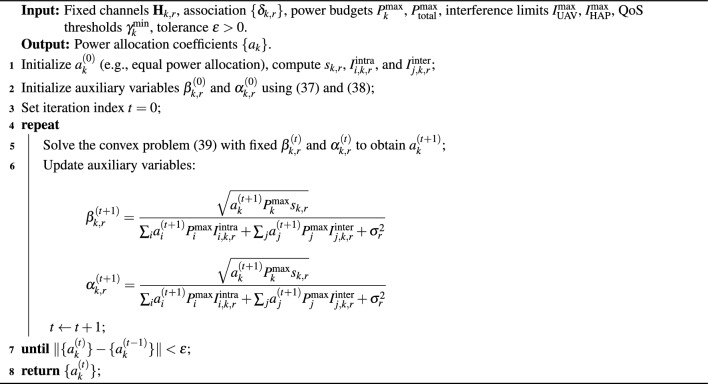
Power Allocation via Quadratic Transform.

The quadratic transform algorithm guarantees convergence to a stationary point of the original non-convex problem. Moreover, the convex subproblem at each iteration can be solved efficiently using standard convex optimization solvers, making the overall power allocation algorithm computationally tractable.

### IRS Phase Shift Subproblem

For fixed UAV deployment $\textbf{L}_{\text {UAV}}$, user association $\{\delta _{k,r}\}$, and power allocation $\{a_k\}$, the IRS phase shift subproblem aims to optimize the reflection matrix ${\boldsymbol{\Phi }} = \operatorname {diag}(e^{j\varphi _1}, \dots , e^{j\varphi _M})$ to maximize the sum rate, subject to unit-modulus constraints. The subproblem is:

40a $$\begin{aligned} \max _{{\boldsymbol{\Phi }}} \quad&{\sum }_{k \in \mathscr {K}} {\sum }_{r \in \{\text {UAV},\text {HAP}\}} \delta _{k,r} B \log _2\left( 1 + \textrm{SINR}_{k,r}({\boldsymbol{\Phi }})\right) \quad \text {s.t.} \quad |[{\boldsymbol{\Phi }}]_{m,m}| = 1, \quad \varphi _m \in [0, 2\pi ), \quad \forall m = 1, \dots , M. \end{aligned}$$

Recall that the effective channels $\textbf{H}_{k,r} = \textbf{h}_{k,r} + \textbf{G}_{\text {IRS},r} {\boldsymbol{\Phi }} \textbf{h}_{k,\text {IRS}}$ depend linearly on the IRS phase shifts. Specifically, let $\boldsymbol{\phi }$ $= [e^{j\varphi _1}, \dots , e^{j\varphi _M}]^T$ denote the vector of IRS reflection coefficients. Then, the cascaded channel can be rewritten as:

41 $$\begin{aligned} \textbf{H}_{k,r} = \textbf{h}_{k,r} + \textbf{G}_{\text {IRS},r} \operatorname {diag}(\textbf{h}_{k,\text {IRS}}) {\boldsymbol{\phi }} \triangleq \textbf{h}_{k,r} + \textbf{D}_{k,r} {\boldsymbol{\phi }}, \end{aligned}$$

where $\textbf{D}_{k,r} = \textbf{G}_{\text {IRS},r} \operatorname {diag}(\textbf{h}_{k,\text {IRS}}) \in \mathbb {C}^{N \times M}$. The SINR expression (13) involves quadratic forms of $\textbf{H}_{k,r}$, which become quartic in ${\boldsymbol{\phi }}$, making the problem highly non-convex. To handle this, we adopt the *Riemannian Conjugate Gradient (RCG)* method, which directly optimizes over the complex circle manifold $\mathscr {M} = \{{\boldsymbol{\phi }} \in \mathbb {C}^M: |\phi _m| = 1, \; m = 1, \dots , M\}$. The RCG method treats the unit-modulus constraints as a manifold and performs optimization on this manifold. The key steps are:

1. **Compute the Euclidean gradient** of the objective function with respect to ${\boldsymbol{\phi }}$.
2. **Project the gradient onto the tangent space** of the manifold at the current point.
3. **Compute the search direction** using the conjugate gradient update on the manifold.
4. **Perform a retraction** to map the updated point back onto the manifold.

We first derive the Euclidean gradient. Let $f({\boldsymbol{\phi }}) = {\sum }_{k,r} \delta _{k,r} R_{k,r}({{\boldsymbol{\phi }}})$ denote the objective function. The gradient with respect to ${\boldsymbol{\phi }}^*$ (the conjugate of ${\boldsymbol{\phi }}$) is given by:

42 $$\begin{aligned} \nabla _{{\boldsymbol{\phi }}^*} f = {\sum }_{k,r} \delta _{k,r} \frac{\partial R_{k,r}}{\partial {\boldsymbol{\phi }}^*}. \end{aligned}$$

Using the chain rule and the expression for $R_{k,r} = B \log _2(1 + \textrm{SINR}_{k,r})$, we have:

43 $$\begin{aligned} \frac{\partial R_{k,r}}{\partial {\boldsymbol{\phi }}^*} = \frac{B}{\ln 2} \frac{1}{1 + \textrm{SINR}_{k,r}} \frac{\partial \textrm{SINR}_{k,r}}{\partial {\boldsymbol{\phi }}^*}. \end{aligned}$$

The SINR gradient can be computed by differentiating (13). Let us define:

44 $$\begin{aligned} \mathscr {I}_{k,r}({\boldsymbol{\phi }})&= {\sum }_{\begin{array}{c} i \in \mathscr {K}_r \\ i \ne k, \; \Vert \textbf{H}_{i,r}\Vert ^2 < \Vert \textbf{H}_{k,r}\Vert ^2 \end{array}} P_i \frac{|\textbf{H}_{k,r}^H \textbf{H}_{i,r}|^2}{\Vert \textbf{H}_{k,r}\Vert ^2} + {\sum }_{j \in \mathscr {K}_{\bar{r}}} P_j \frac{|\textbf{H}_{k,r}^H \textbf{H}_{j,r}|^2}{\Vert \textbf{H}_{k,r}\Vert ^2} + \sigma _r^2, \end{aligned}$$

45 $$\begin{aligned} \mathscr {S}_{k,r}({\boldsymbol{\phi }})&= P_k \Vert \textbf{H}_{k,r}\Vert ^2. \end{aligned}$$

Then $\textrm{SINR}_{k,r} = \mathscr {S}_{k,r} / \mathscr {I}_{k,r}$, and its gradient is: $\frac{\partial \textrm{SINR}_{k,r}}{\partial {\boldsymbol{\phi }}^*} = \frac{1}{\mathscr {I}_{k,r}} \frac{\partial \mathscr {S}_{k,r}}{\partial {\boldsymbol{\phi }}^*} - \frac{\mathscr {S}_{k,r}}{\mathscr {I}_{k,r}^2} \frac{\partial \mathscr {I}_{k,r}}{\partial {\boldsymbol{\phi }}^*}.$ The gradients of $\mathscr {S}_{k,r}$ and $\mathscr {I}_{k,r}$ involve derivatives of quadratic forms. Using the identity $\frac{\partial \textbf{x}^H \textbf{A} \textbf{x}}{\partial \textbf{x}^*} = \textbf{A} \textbf{x}$ for a Hermitian matrix $\textbf{A}$, and noting that $\Vert \textbf{H}_{k,r}\Vert ^2 = \textbf{H}_{k,r}^H \textbf{H}_{k,r}$, we have:

46 $$\begin{aligned} \frac{\partial \mathscr {S}_{k,r}}{\partial {\boldsymbol{\phi }}^*} = P_k \frac{\partial (\textbf{H}_{k,r}^H \textbf{H}_{k,r})}{\partial {\boldsymbol{\phi }}^*} = P_k \left( \frac{\partial \textbf{H}_{k,r}^H}{\partial {\boldsymbol{\phi }}^*} \textbf{H}_{k,r} + \textbf{H}_{k,r}^H \frac{\partial \textbf{H}_{k,r}}{\partial {\boldsymbol{\phi }}^*} \right) . \end{aligned}$$

From (41), $\textbf{H}_{k,r} = \textbf{h}_{k,r} + \textbf{D}_{k,r} {\boldsymbol{\phi }}$, so $\frac{\partial \textbf{H}_{k,r}}{\partial {\boldsymbol{\phi }}^*} = \textbf{0}$ and $\frac{\partial \textbf{H}_{k,r}^H}{\partial {\boldsymbol{\phi }}^*} = \textbf{D}_{k,r}^H$. Therefore, $\frac{\partial \mathscr {S}_{k,r}}{\partial {\boldsymbol{\phi }}^*} = P_k \textbf{D}_{k,r}^H \textbf{H}_{k,r}.$ The gradient of $\mathscr {I}_{k,r}$ is more involved because it includes cross terms. However, each term in $\mathscr {I}_{k,r}$ is of the form $P_i \frac{|\textbf{H}_{k,r}^H \textbf{H}_{i,r}|^2}{\Vert \textbf{H}_{k,r}\Vert ^2}$. Define: $q_{i,k,r} = \frac{\textbf{H}_{k,r}^H \textbf{H}_{i,r}}{\Vert \textbf{H}_{k,r}\Vert }.$ Then $|\textbf{H}_{k,r}^H \textbf{H}_{i,r}|^2 / \Vert \textbf{H}_{k,r}\Vert ^2 = |q_{i,k,r}|^2$. The gradient of $|q_{i,k,r}|^2$ with respect to ${\boldsymbol{\phi }}^*$ is:

47 $$\begin{aligned} \frac{\partial |q_{i,k,r}|^2}{\partial {\boldsymbol{\phi }}^*} = q_{i,k,r} \frac{\partial q_{i,k,r}^*}{\partial {\boldsymbol{\phi }}^*} + q_{i,k,r}^* \frac{\partial q_{i,k,r}}{\partial {\boldsymbol{\phi }}^*} = 2 \Re \left( q_{i,k,r}^* \frac{\partial q_{i,k,r}}{\partial {\boldsymbol{\phi }}^*} \right) . \end{aligned}$$

Using the quotient rule and the expressions for $\textbf{H}_{k,r}$, we can compute $\frac{\partial q_{i,k,r}}{\partial {\boldsymbol{\phi }}^*}$. In practice, we compute the gradient numerically or use automatic differentiation to avoid the complex derivations. For efficiency, we can also approximate the gradient by ignoring the dependence of the denominator $\Vert \textbf{H}_{k,r}\Vert$ on ${\boldsymbol{\phi }}$ when the channel gains are relatively stable, leading to a simplified gradient. Once the Euclidean gradient $\nabla _{{\boldsymbol{\phi }}^*} f$ is obtained, we project it onto the tangent space of the manifold at ${\boldsymbol{\phi }}^{(t)}$. The tangent space at ${\boldsymbol{\phi }}$ is: $T_{{\boldsymbol{\phi }}} \mathscr {M} = \{ \textbf{v} \in \mathbb {C}^M: \Re (\textbf{v} \odot {\boldsymbol{\phi }}^*) = \textbf{0} \},$ where $\odot$ denotes element-wise multiplication. The projection of a Euclidean gradient $\textbf{g}$ onto $T_{{\boldsymbol{\phi }}} \mathscr {M}$ is:

48 $$\begin{aligned} \textrm{Proj}_{{\boldsymbol{\phi }}}(\textbf{g}) = \textbf{g} - \Re (\textbf{g} \odot {\boldsymbol{\phi }}^*) \odot {\boldsymbol{\phi }}. \end{aligned}$$

Let $\boldsymbol{\eta }^{(t)} = \textrm{Proj}_{{\boldsymbol{\phi }}^{(t)}}(\nabla _{{\boldsymbol{\phi }}^*} f)$ denote the Riemannian gradient at iteration *t*. The conjugate gradient direction $\boldsymbol{\xi }^{(t)}$ is updated as: $\boldsymbol{\xi }^{(t)} = -\boldsymbol{\eta }^{(t)} + \mu ^{(t)} \textrm{Proj}_{{\boldsymbol{\phi }}^{(t)}}(\boldsymbol{\xi }^{(t-1)}),$ where $\mu ^{(t)}$ is the Polak–Ribière parameter:

49 $$\begin{aligned} \mu ^{(t)} = \frac{\langle \boldsymbol{\eta }^{(t)}, \boldsymbol{\eta }^{(t)} - \textrm{Proj}_{{\boldsymbol{\phi }}^{(t)}}(\boldsymbol{\eta }^{(t-1)}) \rangle }{\langle \boldsymbol{\eta }^{(t-1)}, \boldsymbol{\eta }^{(t-1)} \rangle }. \end{aligned}$$

Then, we update ${\boldsymbol{\phi }}$ along the direction $\boldsymbol{\xi }^{(t)}$ with a step size $\alpha ^{(t)}$ determined by Armijo backtracking line search on the manifold. The retraction operation maps the updated point back to the manifold:

50 $$\begin{aligned} {\boldsymbol{\phi }}^{(t+1)} = \textrm{Retraction}_{{\boldsymbol{\phi }}^{(t)}}(\alpha ^{(t)} \boldsymbol{\xi }^{(t)}) = \frac{{\boldsymbol{\phi }}^{(t)} + \alpha ^{(t)} \boldsymbol{\xi }^{(t)}}{|{\boldsymbol{\phi }}^{(t)} + \alpha ^{(t)} \boldsymbol{\xi }^{(t)}|}, \end{aligned}$$

where the division is element-wise. The RCG algorithm for IRS phase shift optimization is summarized in Algorithm 4.

**Algorithm 4:**
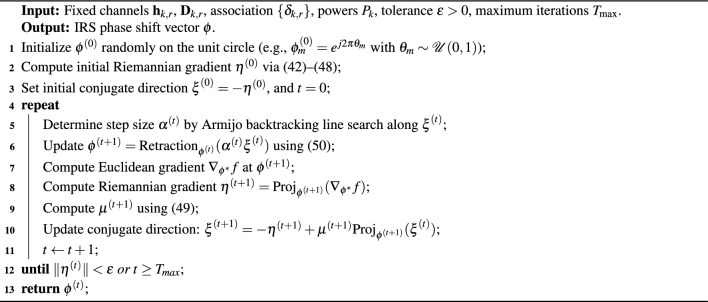
IRS Phase Shift Optimization via Riemannian Conjugate Gradient.

The RCG method efficiently exploits the manifold structure of the unit-modulus constraints and converges to a locally optimal solution with a superlinear rate in practice. Its computational complexity per iteration is linear in the number of IRS elements *M*, making it scalable for large IRS arrays.

### Complexity and convergence analysis

#### Computational complexity

The BCD algorithm’s complexity is dominated by iterative updates of four subproblems. Let $T_1$–$T_4$ denote average inner iterations per subproblem and $T_{\text {BCD}}$ the outer iterations.

**UAV deployment:** SCA solves an Linear Program (LP) with $\mathscr {O}(K^{3.5} + K(N+M))$ per iteration.

**User Association:** Fixed interference approximation costs $\mathscr {O}(K^2N + K\log K)$.

**Power Allocation:** Quadratic transform yields $\mathscr {O}(K^{3.5} + K^2N)$ per iteration.

**IRS Phase Shifts:** RCG requires $\mathscr {O}(K^2NM + KM^2)$ per iteration.

Total complexity is $T_{\text {BCD}} {\sum }_{i=1}^4 T_i \cdot \mathscr {C}_i$, where $\mathscr {C}_i$ are the above complexities. For $M \gg N> K$, IRS optimization dominates, but the algorithm remains practical (typically $T_{\text {BCD}} \le 20$).

#### Signaling overhead

Executing the centralized BCD-IRS-HU algorithm necessitates control signaling to acquire global CSI at the central processing node (e.g., the HAP) and to distribute the optimized resource allocation parameters^36^. For uplink CSI acquisition, acquiring the direct channels ($\textbf{h}_{k,\text {UAV}}, \textbf{h}_{k,\text {HAP}}$), user-to-IRS channels ($\textbf{h}_{k,\text {IRS}}$), and static IRS-to-platform channels ($\textbf{G}_{\text {IRS},\text {UAV}}, \textbf{G}_{\text {IRS},\text {HAP}}$) requires exchanging $2KN + KM + 2MN$ complex scalars per channel coherence block^37^. Since the aerial IRS-to-platform channels are quasi-static, the dynamic uplink overhead reduces to $\mathscr {O}(K(N+M))$. For downlink control, broadcasting the optimized variables, namely the UAV 3D coordinates (3 real scalars), user association (*K* bits), power coefficients (*K* scalars), and IRS phase shifts (*M* scalars), incurs an overhead scaling as $\mathscr {O}(K + M)$. Overall, the signaling overhead scales linearly with *K* and *M*, and can be efficiently managed via the dedicated inter-aerial control link without overwhelming the shared data spectrum.

#### Convergence

##### Proposition 0.2

The BCD algorithm converges to a stationary point of problem (14).

##### Proof

The objective is bounded above and non-decreasing with each block update. Each subproblem converges to its stationary point (SCA for UAV, quadratic transform for power, RCG for IRS, exact for association). By BCD convergence for non-convex block-separable problems, the overall algorithm converges to a stationary point.$\square$

### Joint Algorithm: BCD-IRS-HU

Algorithm 5 summarizes the complete BCD-IRS-HU (Block Coordinate Descent for IRS-assisted HAP-UAV) framework. It alternates between optimizing UAV placement (Algorithm 1), user association (Algorithm 2), power allocation (Algorithm 3), and IRS phase shifts (Algorithm 4) until convergence.

**Algorithm 5:**
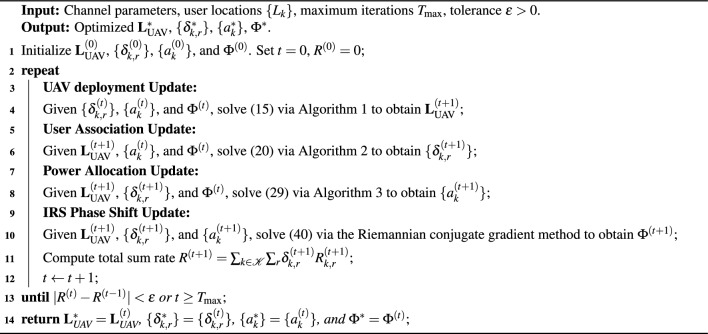
BCD-IRS-HU: Joint Optimization of UAV deployment, User Association, Power Allocation, and IRS Phase Shifts.

Table 1 summarizes the per-iteration complexity. The algorithm typically converges within 10–20 outer iterations, achieving favorable performance-complexity tradeoffs for practical emergency networks.

**Table 1:** Computational Complexity of BCD-IRS-HU Subproblems.

**Subproblem**	**Complexity**	**Subproblem**	**Complexity**
UAV deployment	$\mathscr {O}(K^{3.5} + K(N+M))$	Power Allocation	$\mathscr {O}(K^{3.5} + K^2N)$
User Association	$\mathscr {O}(K^2N + K\log K)$	IRS Phase Shifts	$\mathscr {O}(K^2NM + KM^2)$

## Results and discussion

This section evaluates the performance of the proposed IRS-assisted HAP–UAV uplink NOMA framework through extensive numerical simulations and compares it with several representative benchmark schemes (The reported sum-rate results are obtained under commonly adopted idealized assumptions, including perfect SIC, continuous IRS phase shifts, and perfect CSI. These assumptions provide an upper-bound performance benchmark and offer insights into the potential gains of the proposed framework. The impact of practical impairments, such as discrete phase shifts and imperfect SIC, is left for future investigation.). Unless otherwise stated, all results are obtained under a shared-spectrum uplink setting in which all users are simultaneously scheduled over the same time–frequency resources, and uplink NOMA with SIC is employed at the aerial receivers.

### Simulation setup and compared schemes

We consider a hybrid aerial communication network consisting of one HAP and one UAV, deployed to serve single-antenna ground users jointly. The set of simultaneously active users is denoted by $\mathscr {K}$, with cardinality $|\mathscr {K}|=K$. An IRS equipped with *M* passive reflecting elements is deployed to assist uplink transmission by reconfiguring the wireless propagation environment. All users transmit concurrently in the uplink using power-domain NOMA, and SIC is performed at both the HAP and UAV receivers.

The proposed scheme, referred to as *BCD-IRS-HU*, jointly optimizes the UAV 3D position $\boldsymbol{L}_{\textrm{UAV}}$, user association variables, uplink transmit power allocation, and IRS phase shift matrix using a BCD framework. This joint design explicitly accounts for uplink NOMA decoding order, residual SIC interference, and cross-tier interference arising from spectrum sharing between the HAP and UAV layers.

For performance comparison, the following benchmark schemes are considered:

- **Hybrid HAP–UAV uplink NOMA (no IRS):** Same architecture as the proposed scheme, but without IRS assistance.
- **UAV-only uplink NOMA (with/without IRS):** Users are associated exclusively with the UAV, with/without IRS support.
- **HAP-only uplink NOMA (with/without IRS):** All users are served solely by the HAP, with or without IRS assistance.
- **Terrestrial uplink baseline:** Conventional ground-based uplink transmission without aerial platforms or IRS.

Performance is evaluated in terms of achievable uplink sum rate, average per-user rate, outage probability, convergence behavior, and the Cumulative Distribution Function (CDF) of the uplink sum rate.

**Table 2:** Simulation Parameters^15,38^.

**Parameter**	**Value**	**Parameter**	**Value**
*System Configuration*		*Node Positions [m]*	
Number of users (*K*)	50	Initial UAV pos. ($\boldsymbol{L}_{\textrm{UAV}}^{(0)}$)	[0, 0, 100]
Number of UAVs ($N_{\textrm{UAV}}$)	1	HAP position ($\boldsymbol{L}_{\textrm{HAP}}$)	[500, 500, 300]
Number of HAPs ($N_{\textrm{HAP}}$)	1	IRS position ($\boldsymbol{L}_{\textrm{IRS}}$)	$[10]$
IRS elements (*M*)	100	Simulation area	$1 \times 1$ km
*Channel Model*		*Power & Interference*	
Carrier freq. ($f_c$)	2 GHz	Max. transmit power ($P_k^{\max }$)	23 dBm
Bandwidth (*B*)	10 MHz	UAV recv. limit ($P_{\textrm{UAV}}^{\max }$)	30 dBm
LOS PL exponent ($\alpha _{\textrm{L}}$)	2.0	HAP recv. limit ($P_{\textrm{HAP}}^{\max }$)	30 dBm
NLOS PL exponent ($\alpha _{\textrm{N}}$)	3.5	Interf. threshold (UAV)	$-70$ dBm
LOS shad. var. ($\sigma _{\textrm{L}}^2$)	2 dB	Interf. threshold (HAP)	$-70$ dBm
NLOS shad. var. ($\sigma _{\textrm{N}}^2$)	5 dB	Min. SINR ($\gamma _k^{\min }$)	0 dB
*Optimization Settings*		*Initialization*	
Max. BCD iter. ($T_{\max }$)	100	IRS phase shifts	Random
Conv. tolerance ($\epsilon$)	$10^{-3}$	Power allocation	Equal

Table 2 summarizes the simulation parameters used throughout the numerical evaluation. A single HAP and a single UAV are considered to clearly isolate the performance gains enabled by hybrid aerial deployment and IRS assistance. Unless otherwise specified, $K=50$ users are simultaneously scheduled to represent a moderately dense uplink scenario. The IRS is equipped with $M=100$ passive reflecting elements, which provide sufficient spatial resolution to demonstrate effective passive beamforming gains without incurring excessive hardware complexity. A carrier frequency of 2 GHz and a bandwidth of 10 MHz are adopted to reflect a practical sub-6 GHz deployment suitable for wide-area aerial coverage. The channel model accounts for both LOS and NLOS propagation with distinct PL exponents and log-normal shadowing, capturing realistic air-to-ground and air-to-air channel characteristics. The HAP, UAV, and IRS are placed at representative altitudes to ensure high LOS probability while allowing meaningful cross-tier interference interactions. Each user is subject to a maximum uplink transmit power of 23 dBm. At the same time, the aerial platforms are constrained by receive-power and interference thresholds to regulate cross-tier interference in the shared-spectrum uplink explicitly. The proposed BCD-based optimization is executed with a maximum of 100 iterations and a convergence tolerance of $10^{-3}$, ensuring reliable convergence with manageable computational complexity. Random IRS phase initialization and equal-power allocation are used as unbiased starting points, ensuring that all reported performance gains stem from the proposed joint optimization.

**Fig. 2:**
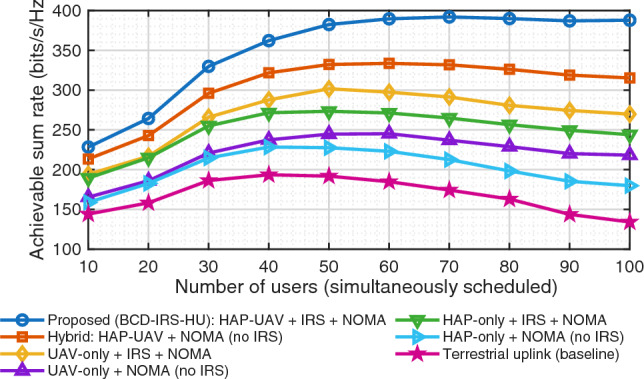
Achievable uplink sum rate versus the number of simultaneously scheduled users.

**Fig. 3:**
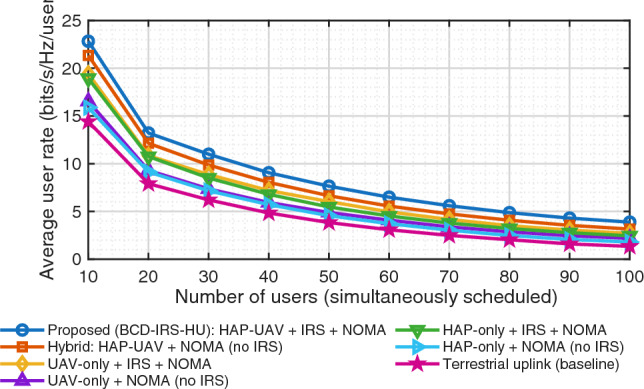
Average per-user uplink rate versus the number of simultaneously scheduled users.

### Impact of the number of simultaneously scheduled users

Figures 2 and 3 show the uplink performance as the number of simultaneously scheduled users increases. As all users share the same time–frequency resources, both uplink NOMA intra-tier interference and HAP–UAV cross-tier interference intensify with user density. **Sum-rate performance (Fig.** 2**):** The proposed IRS-assisted HAP–UAV uplink NOMA framework achieves the highest sum rate across all user loads. Compared to the hybrid HAP–UAV scheme without IRS, sum-rate gains of $15.13\%$, $16.79\%$, and $23.03\%$ are achieved at $K=50$, 60, and 100, respectively, with an average improvement of $15.39\%$ over $K=10$–100. Relative to UAV-only transmission with IRS, the gains increase to $26.77\%$ at $K=50$ and $43.80\%$ at $K=100$, while improvements of up to $59.01\%$ are observed over HAP-only transmission with IRS. The proposed framework further outperforms the terrestrial uplink baseline by more than $100\%$ on average and up to $189.11\%$ at high user loads. All schemes exhibit saturation at large *K* due to increased aggregate interference and residual SIC effects. **Per-user rate performance (Fig. **3**):** The average per-user rate decreases with *K* for all schemes, but the proposed framework consistently maintains the highest per-user rate. The relative gains mirror the sum-rate results, confirming that the proposed joint optimization improves both aggregate throughput and scalability by effectively controlling interference. Overall, these results highlight the necessity of a joint interference-aware design for IRS-assisted HAP–UAV uplink NOMA systems, particularly under dense user scheduling.

**Fig. 4:**
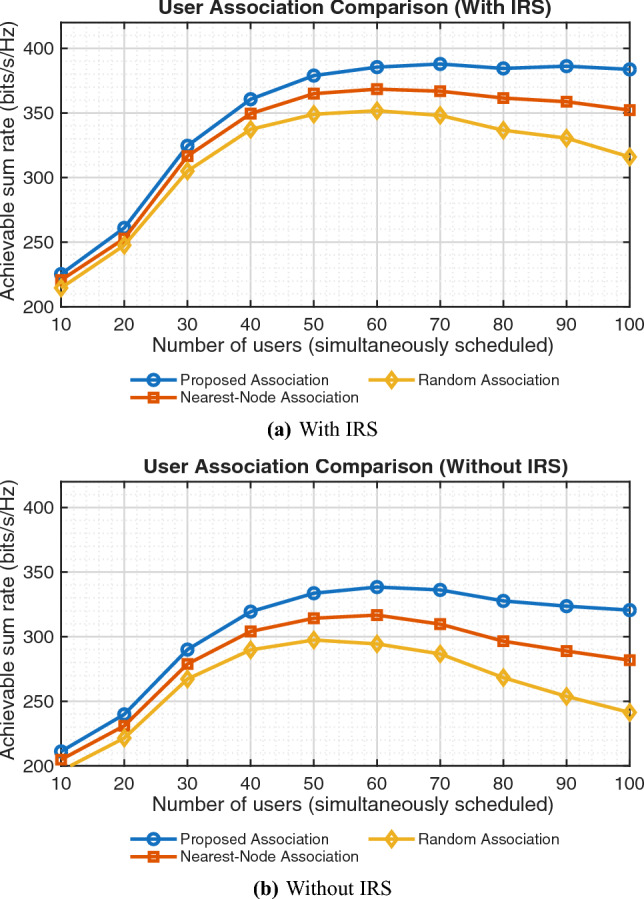
Uplink sum rate under different user association strategies: (**a**) with IRS and (**b**) without IRS.

### Impact of user association under IRS-assisted and non-IRS uplink transmission

Figure 4 shows the uplink sum rate versus the number of simultaneously scheduled users for different user association strategies, including the proposed optimized association, nearest-node association, and random association. In all cases, users share the same time–frequency resources and uplink NOMA with SIC is employed, leading to increasing intra-tier and cross-tier interference as the user load grows.

**IRS-assisted case:** With IRS enabled, the proposed user association consistently achieves the highest uplink sum rate across all user loads. At $K=50$, the proposed strategy provides gains of $3.83\%$ and $8.57\%$ over nearest-node and random association, respectively, which increase to $4.63\%$ and $9.60\%$ at $K=60$, and further to $8.95\%$ and $21.45\%$ at $K=100$. Averaged over $K=10$–100, the proposed association improves the sum rate by $4.82\%$ and $10.59\%$ compared to nearest-node and random strategies, respectively, demonstrating that optimized association remains essential for interference mitigation even with IRS assistance.

**Non-IRS case:** Without IRS, overall performance degrades due to weaker composite channels and reduced interference control, but the relative gains of optimized association become more pronounced. At $K=50$, the proposed strategy outperforms nearest-node and random association by $6.16\%$ and $12.17\%$, respectively, increasing to $6.86\%$ and $14.95\%$ at $K=60$, and reaching $13.75\%$ and $32.82\%$ at $K=100$. On average, improvements of $7.38\%$ and $16.14\%$ are achieved, confirming the higher sensitivity of non-IRS systems to suboptimal association.

These results confirm that optimized user association is a key enabler for scalable uplink performance in shared-spectrum HAP–UAV NOMA systems. While IRS assistance alleviates propagation impairments, intelligent association remains critical to control cross-tier interference and maintain robust performance under dense uplink access.

**Fig. 5:**
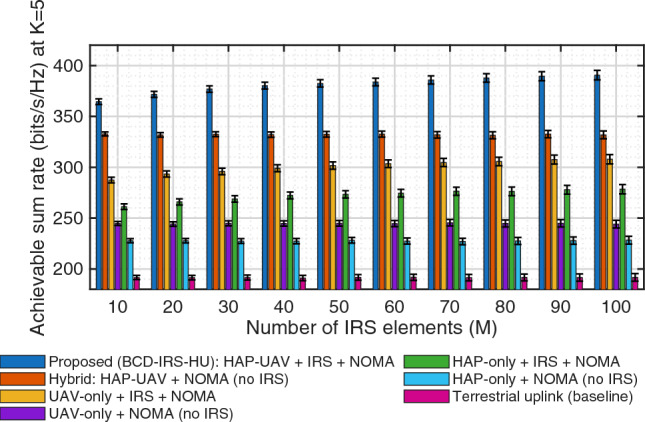
Achievable uplink sum rate versus the number of IRS elements *M* at a fixed number of simultaneously scheduled users ($K=50$).

### Impact of the number of IRS elements

Fig. 5 shows the achievable uplink sum rate versus the number of IRS elements *M* for a fixed user load of $K=50$. For all IRS-enabled schemes, the sum rate increases with *M* due to enhanced passive beamforming gain and improved effective channel strength, while non-IRS schemes remain insensitive to *M*. The proposed IRS-assisted HAP–UAV uplink NOMA framework consistently achieves the highest performance. Compared to the hybrid HAP–UAV uplink NOMA scheme without IRS, it provides sum-rate gains of $9.56\%$, $15.05\%$, and $17.70\%$ at $M=10$, 50, and 100, respectively, with an average improvement of $14.80\%$. Relative to UAV-only IRS-assisted uplink NOMA, the proposed scheme achieves a nearly constant gain of about $26.8\%$ across all *M*, indicating that the primary performance advantage stems from the dual-layer HAP–UAV architecture rather than IRS scaling alone. Compared to HAP-only IRS-assisted uplink NOMA, the proposed framework yields gains of approximately $40\%$ on average, highlighting the importance of UAV-assisted spatial reuse. The largest relative improvement is observed over the terrestrial uplink baseline, where the proposed scheme achieves gains of $99.59\%$ at $M=50$ and $103.78\%$ at $M=100$, with an average gain of $99.12\%$. These results confirm that IRS deployment is most effective when jointly integrated with a hybrid HAP–UAV architecture and interference-aware uplink NOMA transmission, while increasing *M* alone exhibits diminishing returns at large IRS sizes.

**Fig. 6:**
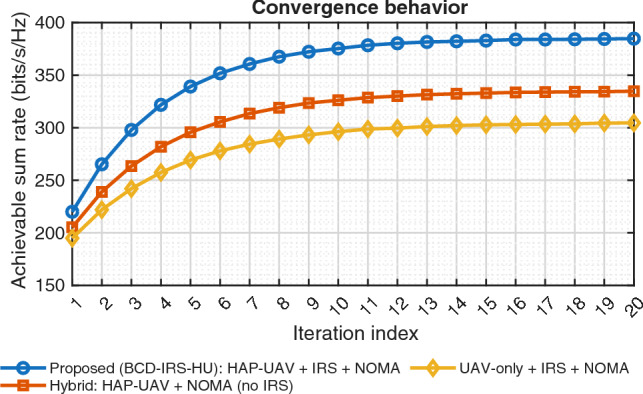
Convergence of the BCD-IRS-HU algorithm in terms of uplink sum rate ($K=50$).

### Convergence behavior

Fig. 6 shows the convergence of the proposed BCD-IRS-HU framework. The sum rate increases rapidly in the first few iterations due to the largest gains from updating the strongly coupled variables (gains from UAV deployment, association, power control, and IRS phases). It then gradually saturates as the algorithm approaches a stationary point. The proposed scheme converges to the highest final sum rate. In contrast, the benchmark schemes converge to lower plateaus because they lack either IRS-based channel reconfiguration and/or the dual-layer HAP–UAV flexibility. This behavior is consistent with block coordinate descent: each iteration yields a non-decreasing objective improvement, and diminishing returns appear near convergence.

**Fig. 7:**
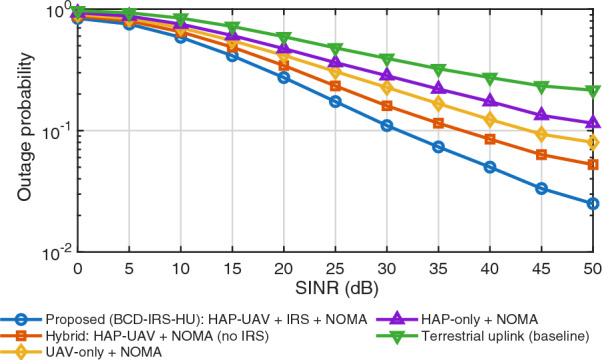
Outage probability versus SINR for the proposed IRS-assisted HAP–UAV uplink NOMA scheme and benchmarks.

### Outage performance versus SINR

Fig. 7 shows the outage probability as a function of the SINR threshold. As the threshold increases, the outage probability decreases for all schemes because fewer channel realizations fall below the required SINR. The proposed IRS-assisted HAP–UAV uplink NOMA scheme achieves the lowest outage over the entire SINR range, since (i) the dual-layer HAP–UAV architecture provides stronger links via spatial reuse and traffic offloading, and (ii) the IRS improves the composite channels by enhancing desired signal components and partially suppressing interference.

Compared to the hybrid HAP–UAV scheme without IRS, the proposed design consistently shifts the outage curve downward, indicating improved reliability due to propagation-environment reconfiguration. The UAV-only scheme generally outperforms the HAP-only scheme because of shorter air-to-ground distances and stronger average channels. At the same time, the terrestrial baseline exhibits the highest outage due to the absence of aerial LOS advantages and weaker propagation conditions. Overall, the figure confirms that combining IRS assistance with a hybrid HAP–UAV uplink architecture is effective in reducing outage and improving reliability under interference-limited massive uplink access.

**Fig. 8:**
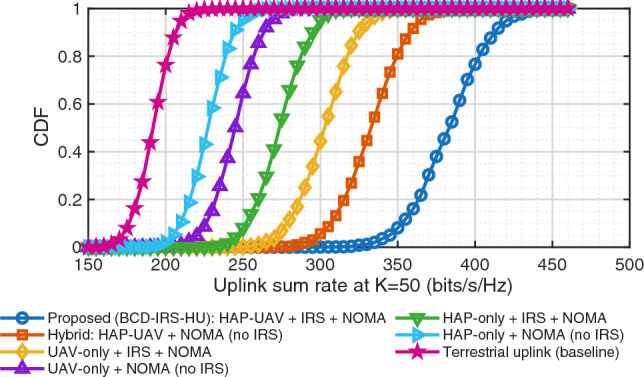
CDF of the uplink sum rate at a fixed user load of $K=50$ for different transmission architectures.

### CDF analysis of uplink sum rate

Fig. 8 presents the CDF of the uplink sum rate at $K=50$ simultaneously scheduled users, which provides a reliability-oriented view of system performance beyond average metrics. A rightward shift of a CDF curve indicates a higher probability of achieving larger sum rates.

The proposed IRS-assisted HAP–UAV uplink NOMA scheme exhibits the most favorable distribution, with its CDF consistently shifted to the right of all benchmark schemes. This indicates not only a higher mean sum rate but also improved robustness against channel variations. For example, at a target outage level of $\textrm{CDF}=0.5$, the proposed scheme achieves a sum rate of approximately 400 bits/s/Hz, whereas hybrid HAP–UAV without IRS, UAV-only with IRS, and HAP-only with IRS achieve roughly 350, 300, and 260 bits/s/Hz, respectively. The terrestrial baseline shows the poorest reliability, with most of the probability mass concentrated at low sum rates due to unfavorable propagation conditions.

The clear ordering of the CDF curves is fully consistent with the trends observed in the average sum-rate and per-user rate results. IRS-assisted schemes dominate their non-IRS counterparts, confirming the effectiveness of passive beamforming in enhancing channel quality. Moreover, hybrid HAP–UAV architectures outperform single-layer aerial deployments, highlighting the benefit of spatial diversity and load balancing across aerial tiers. Overall, this CDF-based evaluation confirms that the proposed framework not only maximizes average uplink throughput but also significantly increases the probability of achieving high sum rates, making it particularly suitable for reliable, dense uplink connectivity scenarios.

### Discussion on practical system impairments

The performance gains presented in this study establish a theoretical upper bound predicated on perfect CSI and ideal SIC. In practical deployments, mobility, hardware non-linearities, and signaling latency introduce inevitable channel uncertainties and decoding errors.

**Imperfect CSI **^39^: Due to outdated channel estimation, the true effective uplink channel $\textbf{H}_{k,r}$ is more accurately characterized by a deterministic bounded uncertainty model:

$$\begin{aligned} \textbf{H}_{k,r} = \hat{\textbf{H}}_{k,r} + \Delta \textbf{H}_{k,r}, \quad \forall \Delta \textbf{H}_{k,r} \in \Omega _{k,r} \end{aligned}$$

where $\hat{\textbf{H}}_{k,r}$ is the acquired channel estimate and $\Delta \textbf{H}_{k,r}$ is the CSI error bounded by the uncertainty set $\Omega _{k,r} \triangleq \{ \Delta \textbf{H}_{k,r}: \Vert \Delta \textbf{H}_{k,r}\Vert _F \le \varepsilon _{k,r} \}$, with $\varepsilon _{k,r}$ denoting the maximum error radius.

**Imperfect SIC **^40^: Phase noise and imperfect CSI preclude exact signal subtraction, leading to error propagation. Letting $\varpi _{i} \in [0,1]$ denote the fractional SIC error factor for a previously decoded stronger user *i*, the generalized SINR from Eq. (13) under imperfect SIC becomes:

$$\begin{aligned} \widetilde{\textrm{SINR}}_{k,r} = \frac{P_k \Vert \textbf{H}_{k,r}\Vert ^2}{ \sum \limits _{\begin{array}{c} i\in \mathscr {K}_r \\ \Vert \textbf{H}_{i,r}\Vert ^2 < \Vert \textbf{H}_{k,r}\Vert ^2 \end{array}} P_i \frac{|\textbf{H}_{k,r}^H\textbf{H}_{i,r}\Vert ^2}{\Vert \textbf{H}_{k,r}\Vert ^2} + \sum \limits _{\begin{array}{c} i\in \mathscr {K}_r \\ \Vert \textbf{H}_{i,r}\Vert ^2> \Vert \textbf{H}_{k,r}\Vert ^2 \end{array}} \varpi _{i} P_i \frac{|\textbf{H}_{k,r}^H\textbf{H}_{i,r}\Vert ^2}{\Vert \textbf{H}_{k,r}\Vert ^2} + I_{k,r}^{\textrm{cross}} + \sigma _r^2 } \end{aligned}$$

where $I_{k,r}^{\textrm{cross}}$ encapsulates the cross-tier interference. As $\varpi _{i} \rightarrow 1$, residual intra-cluster interference scales up, inherently compressing the performance gap between NOMA and orthogonal benchmarks.

Integrating these impairments transforms the MINLP in Eq. (14) into a semi-infinite worst-case robust optimization problem of the form $\max _{\mathscr {X}} \min _{\Delta \textbf{H} \in \Omega } f(\mathscr {X}, \Delta \textbf{H})$. While solving this robust max-min formulation introduces significant computational intractability beyond the scope of this baseline framework, developing a worst-case or chance-constrained extension of the BCD-IRS-HU algorithm is a primary objective for future work.

## Conclusion

This paper proposed an IRS-assisted hybrid HAP–UAV uplink NOMA framework to support dense and interference-limited uplink transmission under shared-spectrum operation. By jointly modeling UAV 3D deployment, IRS-enhanced composite channels, SIC-based uplink NOMA reception, and cross-tier interference, a comprehensive uplink system model was developed. A joint uplink sum-rate maximization problem was formulated under practical mobility, power, and interference constraints and efficiently solved using a low-complexity BCD framework. Simulation results demonstrated that the proposed framework consistently outperforms conventional benchmarks across multiple metrics. Compared to hybrid HAP–UAV uplink NOMA without IRS, the proposed scheme achieves uplink sum-rate improvements of up to $23.03\%$ at high user densities, while gains of up to $43.80\%$ and $59.01\%$ are observed over UAV-only and HAP-only IRS-assisted uplink schemes, respectively. Significant performance advantages are also obtained over terrestrial uplink baselines under dense access conditions. The results further show that interference-aware user association and joint optimization of UAV positioning and IRS phase shifts are critical for enhancing the uplink sum rate, reducing outage probability, and improving rate reliability. While increasing the number of IRS elements yields additional gains, diminishing returns are observed at large IRS sizes, highlighting the importance of system-level joint optimization. Overall, this work demonstrates that integrating IRS technology with a hybrid HAP–UAV architecture and interference-aware uplink optimization provides a scalable and reliable solution for future 6G uplink networks. By enabling high-performance connectivity in remote and challenging terrains, the proposed framework serves as a key enabler for digitalized mining operations and automated safety monitoring, directly supporting the industrial transformation goals of Saudi Vision 2030. Future research directions include multi-UAV extensions, imperfect CSI, dynamic traffic models, and learning-assisted real-time optimization. Furthermore, transitioning this theoretical framework to real-world deployments necessitates addressing practical challenges in IRS deployment. Environmental constraints, such as the availability of suitable building facades and physical blockages, can severely restrict optimal IRS positioning. Additionally, potential platform mobility introduces dynamic alignment and beam-tracking complexities, while the real-time control overhead required to update massive IRS phase-shift matrices continuously remains a critical network bottleneck. Overcoming these physical and signaling limitations to ensure real-world applicability will be a primary focus of our future investigations.

## Data Availability

The datasets used and/or analysed during the current study are available from the corresponding author on reasonable request.
